# Joint MiRNA/mRNA Expression Profiling Reveals Changes Consistent with Development of Dysfunctional Corpus Luteum after Weight Gain

**DOI:** 10.1371/journal.pone.0135163

**Published:** 2015-08-10

**Authors:** Andrew P. Bradford, Kenneth Jones, Katerina Kechris, Justin Chosich, Michael Montague, Wesley C. Warren, Margaret C. May, Zain Al-Safi, Satu Kuokkanen, Susan E. Appt, Alex J. Polotsky

**Affiliations:** 1 Department of Obstetrics & Gynecology, University of Colorado School of Medicine, Aurora, CO 80045, United States of America; 2 Department of Biochemistry, University of Colorado School of Medicine, Aurora, CO 80045, United States of America; 3 Department of Biostatistics and Informatics, Colorado School of Public Health, University of Colorado Denver, Aurora, CO 80045, United States of America; 4 The Genome Institute, Washington University School of Medicine, St Louis, MO 63108, United States of America; 5 Department of Pathology (Comparative Medicine), Wake Forest University Primate Center, Winston-Salem, NC 27157, United States of America; 6 Albert Einstein College of Medicine, Montefiore Medical Center, Bronx, NY 10461, United States of America; China Agricultural University, CHINA

## Abstract

Obese women exhibit decreased fertility, high miscarriage rates and dysfunctional corpus luteum (CL), but molecular mechanisms are poorly defined. We hypothesized that weight gain induces alterations in CL gene expression. RNA sequencing was used to identify changes in the CL transcriptome in the vervet monkey (*Chlorocebus aethiops*) during weight gain. 10 months of high-fat, high-fructose diet (HFHF) resulted in a 20% weight gain for HFHF animals vs. 2% for controls (p = 0.03) and a 66% increase in percent fat mass for HFHF group. Ovulation was confirmed at baseline and after intervention in all animals. CL were collected on luteal day 7–9 based on follicular phase estradiol peak. 432 mRNAs and 9 miRNAs were differentially expressed in response to HFHF diet. Specifically, miR-28, miR-26, and let-7b previously shown to inhibit sex steroid production in human granulosa cells, were up-regulated. Using integrated miRNA and gene expression analysis, we demonstrated changes in 52 coordinately regulated mRNA targets corresponding to opposite changes in miRNA. Specifically, 2 targets of miR-28 and 10 targets of miR-26 were down-regulated, including genes linked to follicular development, steroidogenesis, granulosa cell proliferation and survival. To the best of our knowledge, this is the first report of dietary-induced responses of the ovulating ovary to developing adiposity. The observed HFHF diet-induced changes were consistent with development of a dysfunctional CL and provide new mechanistic insights for decreased sex steroid production characteristic of obese women. MiRNAs may represent novel biomarkers of obesity-related subfertility and potential new avenues for therapeutic intervention.

## Introduction

Over 300 million adult women are classified as obese worldwide [1]. Female obesity is frequently associated with ovulatory and menstrual dysfunction [2], increased congenital anomalies [3], as well as iatrogenic [4] and spontaneous preterm birth [5]. Adiposity exerts a harmful impact on reproductive function even if ovulatory capacity is preserved. Obese women with spontaneous and regular ovulation demonstrate increased pregnancy loss [6, 7], longer time to pregnancy [8], and decreased fertility [9]. Maternal obesity is also an independent risk factor for fetal origin of adult disease, including obesity and metabolic disorders in offspring [10]. Mechanisms for obesity-linked reproductive dysfunction may hold the key to avoiding its transgenerational impact and have considerable public health implications.

Obese women exhibit a state of relative hypogonadotropic hypogonadism [11–14]. Both pituitary and ovarian markers are affected as evidenced by reduced output of luteinizing hormone (LH) and luteal progesterone [13, 15]. In a large-scale evaluation of daily hormone patterns from ovulatory cycles, obese women exhibited greater than 50% decrease in luteal progesterone output and a 30% reduction in LH, when compared to their normal weight counterparts [16]. This implies that the ovary undergoes additional dysfunction in excess of that occurring at the hypothalamus and pituitary, yet molecular mechanisms remain unclear.

MicroRNAs (miRNAs) are non-coding RNAs that function post-transcriptionally [17] and are estimated to regulate up to 30% of all protein-coding genes [18]. In 2008, impairment of miRNA processing was demonstrated as a cause of corpus luteum (CL) insufficiency and infertility in mice [19]. Several more recent studies have shown that miRNA-mediated control of mRNA transcripts is critical for ovarian function [20–22]. Because of the diversity of miRNA targeting, identification of functionally important mRNA targets is crucial for recognizing tissue-specific roles for miRNAs [23]. Several miRNAs regulate ovarian sex steroid synthesis in vitro [24]. The role of miRNAs in mediating effects of adiposity on CL function has not been explored.

The vervet monkey (*Chlorocebus aethiops sabaeus*) is an Old World, nonhuman primate of the same subfamily as macaques. Adult females have an average intermenstrual interval of 30 days and exhibit cyclic hormonal changes that mimic women [25–28]. In a fully pedigreed and genotyped vervet research colony (VRC) of over 400 animals [29], vervets developed obesity and its associated metabolic profile in a manner very similar to humans [30]. Thus, the VRC represents a source of a potentially highly translatable primate model to elucidate the impact of obesity on various target organs, including the reproductive axis. We hypothesized that there are alterations in vervet CL gene expression that occur due to adiposity. MiRNA and mRNA differential expression patterns were compared between monkeys receiving either a high-fat, high-fructose (HFHF) or control diets.

We have documented changes in the CL gene expression in response to diet-induced weight gain. To the best of our knowledge, this is the first report of dietary-induced responses of the ovulating ovary to developing adiposity. Use of a menstrual non-human primate species allowed us to apply invasive investigative tools that cannot be practically used in humans. Thus, our work bridges a knowledge gap by addressing potential underlying molecular mechanisms for effects of obesity on ovarian function.

## Materials and Methods

### Animal Handling, Morphometrics and Urine collection

Ten adult female vervet monkeys (*Chlorocebus aethiops sabaeus*) were selected randomly from the middle of distribution of body mass from the Vervet Research Colony at the Wake Forest Primate Center (WFRC, Winston-Salem, NC). Monkeys were pair-housed at Wake Forest School of Medicine Primate Center / Center for Comparative Medicine and Research/ Friedberg Campus (Winston-Salem, NC). Monkeys were housed indoors in a climate controlled, temperature and humidity monitored room/building. The caging was USDA approved, steel Quad cages constructed of mesh flooring, removable dividers to allow horizontal movement between the two cages for each pair of monkeys, and a pan underneath to collect excrement and other waste. Monkeys were exposed to artificial lighting in a 12 hour, light-dark cycle from 6am to 6pm, with additional ambient light via windows in the hallway external to their housing room. Monkeys had ad-libitum access to water through water lixits and were fed 120kcal of experimental diet /kg of body weight once per day, as detailed in SI Materials and Methods. In addition, feeding and foraging opportunities were provided 3–4 times per week (fresh fruits & vegetables, popcorn, sunflower seeds). For environmental enrichment, all cages were equipped with perches inside and hanging mirrors and puzzle feeders on the outside of the cage. The WFRC Friedberg Campus is an AAALAC-accredited facility and all housing is AAALAC-and FDA approved. All monkeys were within the normal weight range for this species and were sexually mature with a mean age of 6.8 years (Table 1) as detailed further in SI Materials and Methods. This study was carried out in strict accordance with the recommendations in the Guide for the Care and Use of Laboratory Animals of the National Institutes of Health and approved by the WFRC Animal Care and Use Committee. All policies and procedures were done in compliance with state and federal laws, and regulations and guidelines established by the WFRC Animal Care and Use Committee (Protocol number A10-093). Wake Forest University is accredited by the Association for Assessment and Accreditation of Laboratory Animal Care (AAALAC: Assurance number A3391-01). No adverse events occurred. After the study, all animals were examined and released back into the colony.

**Table 1:** Biometric and Metabolic Characteristics Before and After Dietary Intervention*.

Variable		Control Diet (n = 4)	HFHF Diet (n = 6)
Age (years)	Baseline	7.8 (1.2)	6.2 (0.9)
Body weight (kg)	Baseline	5.1 (0.5) ^0.72^	4.4 (0.2) ^0.72^
	10 months	5.3 (0.4) ^0.72^	5.2 (0.2) ^0.02^
	Percent change	2.4 (4.1)	19.6 (7.0) ^0.03^
Waist circumference (cm)	Baseline	28.9 (2.4) ^0.72^	25.4 (1.1)
	10 months	29.0 (1.3) ^0.93^	30.0 (1.2) ^0.03^
	Percent change	1.9 (6.0)	19.0 (6.6) ^0.20^
Total Body Fat Mass (g)	Baseline	999.4 (179.4) ^0.^	682.6 (83.2)
	10 months	1160.4 (129.3) ^0.27^	1310.8 (183.6) ^0.02^
	Percent change	23.1 (17.1)	105.5 (40.5) ^0.07^
Percentage Body Fat	Baseline	19.1 (2.2) ^0.72^	15.1 (1.3)
	10 months	21.5 (1.0) ^0.39^	24.3 (2.7) ^0.02^
	Percent change	16.7(12.0)	65.9 (22.3)^0.11^
Triglycerides (mg/dL)	Baseline	51.3 (3.9) ^0.72^	33.8 (1.6)
	10 months	36.0 (4.5) ^0.09^	68.0 (25.7) ^0.03^
	Percent change	-27.6 (12.1)	104.2 (78.5) ^0.01^
Total cholesterol (mg/dL)	Baseline	168.3 (6.1) ^0.72^	151.2 (10.0)
	10 months	163.0 (7.7) ^0.67^	158.2 (11.1) ^0.42^
	Percent change	-2.5 (6.8)	5.1 (4.8) ^0.39^
Total Adiponectin (ng/mL)	Baseline	49,602 (4931) ^0.72^	57,548 (5568) ^0.72^
	10 months	46,445 (6179) ^0.68^	46,166 (6841) ^0.13^
	Percent change	-4.4 (14.6)	-19.3 (11.8) ^0.44^

Values indicate mean (standard error of mean). HFHF, High fat high fructose

* Superscripts are P values for within the group comparisons for 10 month values (vs. baseline) and for between the group comparisons for percent change values (control vs. HFHF diet)

### Serum and Urine Analytes and Assays

The urinary excretion of sex steroids closely corresponds to the serum concentrations of the parent hormones [31]. Urine was assayed for estrone conjugates (E1c) and pregnanediol glucuronide (Pdg) using previously described methods [32], as detailed further in SI Materials and Methods (S2 Table).

### Luteectomy

In order to time the luteal phase, serum estradiol levels were measured daily from menstrual cycle day 7 during the luteectomy cycle. The first day of low serum estradiol (defined as values less than 100 pg/ml [33]) after the midcycle estradiol peak was denoted day 1 of the luteal phase [27]. Luteectomies were performed twice on all monkeys, at baseline and after dietary intervention. A laparotomy approach was used to collect CL tissue on luteal day 7–9 as detailed further in SI Materials and Methods (S1 Fig).

### Dietary Intervention

At baseline, the monkeys were fed a commercial non-human primate diet (Purina Monkey Chow) once daily in the afternoon. After completion of the baseline procedures, monkeys were randomly assigned to either adipogenic HFHF diet (n = 6), similar to that used for previous studies in baboons [34] and cynomolgus monkeys [35] or a control diet (n = 4) (Table 2) as detailed further in SI Materials and Methods (S1 Table).

**Table 2:** Composition of Control and High Fat High Fructose (HFHF) Experimental Diets.

Diet	Caloric Density	Protein (% Kcal)	Carbohydrate (% Kcal)	Simple sugars (% Kcal)	Fat (% Kcal)	Fiber (% of diet)
Control	2.3^**†**^	17.6	57	11	26	12.8
HFHF	2.7^**†**^	17.1	45	30	38	10.5
Drink	0.60*	0	100	100	0	0

Monkeys were fed 120 Kcal of diet / kg of body weight per day plus 10% to account for waste. Simple sugars were derived from sucrose (3% for both diets) and high fructose corn syrup (HFCS; 2.4% for control and 10% for HFHF). In addition to the diet, monkeys in the HFHF group were given daily access to a Kool-Aid drink containing 15ml of HFCS / 100ml of water, providing 150–250 additional Kcal per day.

^†^ Kcal/g of diet

* Kcal/ml

### RNA isolation, Library Preparation, and Sequencing

CL total RNA was extracted using TRIzol method followed by purification using MirVana RNA Isolation Kit (Ambion) as detailed further in SI Materials and Methods.

### Computational Analysis and Quality Control. mRNA profiling

All sequence reads were trimmed to an overall quality score of Q15 [36], and any sequences that were trimmed to less than 75bp were subsequently removed, as detailed further in SI Materials and Methods (S4 Table).

### miRNA profiling

As the miRNA sequence reads are shorter than the 50bp reads generated, the first step in quality assessment of the miRNA reads was to identify the reverse complement of the reverse sequencing adapter. As no vervet monkey-specific miRNA databases exist, it was necessary to take an agnostic approach to find all small RNAs that might be differentially expressed. Thus reads that passed quality filtering were analyzed with a custom Python script to identify unique sequences, and produce a normalized read count using DESeq normalization [37] as detailed further in SI Materials and Methods (S5 Table).

### Genome Annotation

Transcript sequencing output was mapped with the current vervet genome assembly (Chlorocebus_sabeus 1.0). We utilized the Vervet Genome Sequencing Project [38] as detailed further in SI Materials and Methods.

### Target Gene Prediction and Integrated Analysis

The selected miRNAs that were differentially expressed were further analyzed to identify the networks and pathways targets as detailed further in SI Materials and Methods.

## Results

### Acquisition of Adiposity Following 10 Months of HFHF Diet

Animals were given either HFHF or control diet (Table 1, S1 Table) after baseline studies were completed (S2 Table). Monkeys in the intervention group gained fat mass and body weight, increased their waist circumference, and developed a significant increase in serum triglycerides (Table 2). On average, dietary intervention resulted in a 20% weight gain for HFHF-fed monkeys vs. 2% in controls (p = 0.03). There was a 66% increase in percent body fat for HFHF group vs. 17% for control. While fasting glucose and total cholesterol were not significantly affected, the reduction in total adiponectin (-19%) did approach statistical significance for the experimental group (p = 0.13). Assessment of reproductive hormones showed no differences between the two groups with respect to menses or serum AMH. All animals were ovulatory before and after intervention, based on assays of sex steroid metabolites from serial urine collections. Indices of estrogen excretion and luteal phase progesterone excretion were similar before and after intervention (S2 Table). This implied that our model is best suited to assess early, pre-clinical CL response to *de novo* adiposity.

To evaluate changes in CL gene expression that mediate adiposity-related reduction in reproductive fitness, we performed luteectomy at baseline and after the dietary intervention. For each analyzed cycle, lifespan of CL was timed by daily serum estradiol (E2) in the follicular phase to document midcycle E2 surge and subsequent E2 drop corresponding to LH surge [39]. All luteectomy procedures were conducted on luteal day 7–9 as this corresponds to a mid-stage, fully functioning CL based on dynamic transcript changes during CL developmental phases in the rhesus macaque [33]. RNA sequencing was conducted on the obtained CL tissue by paired assessments of the same animal. Joint genomic profiling of mRNA and miRNA was done to evaluate the initial adaptive changes of the ovulating ovary to weight gain.

### mRNA Expression Changes with Adiposity, Weight Gain and Fat Mass Gain

**Fig 1:**
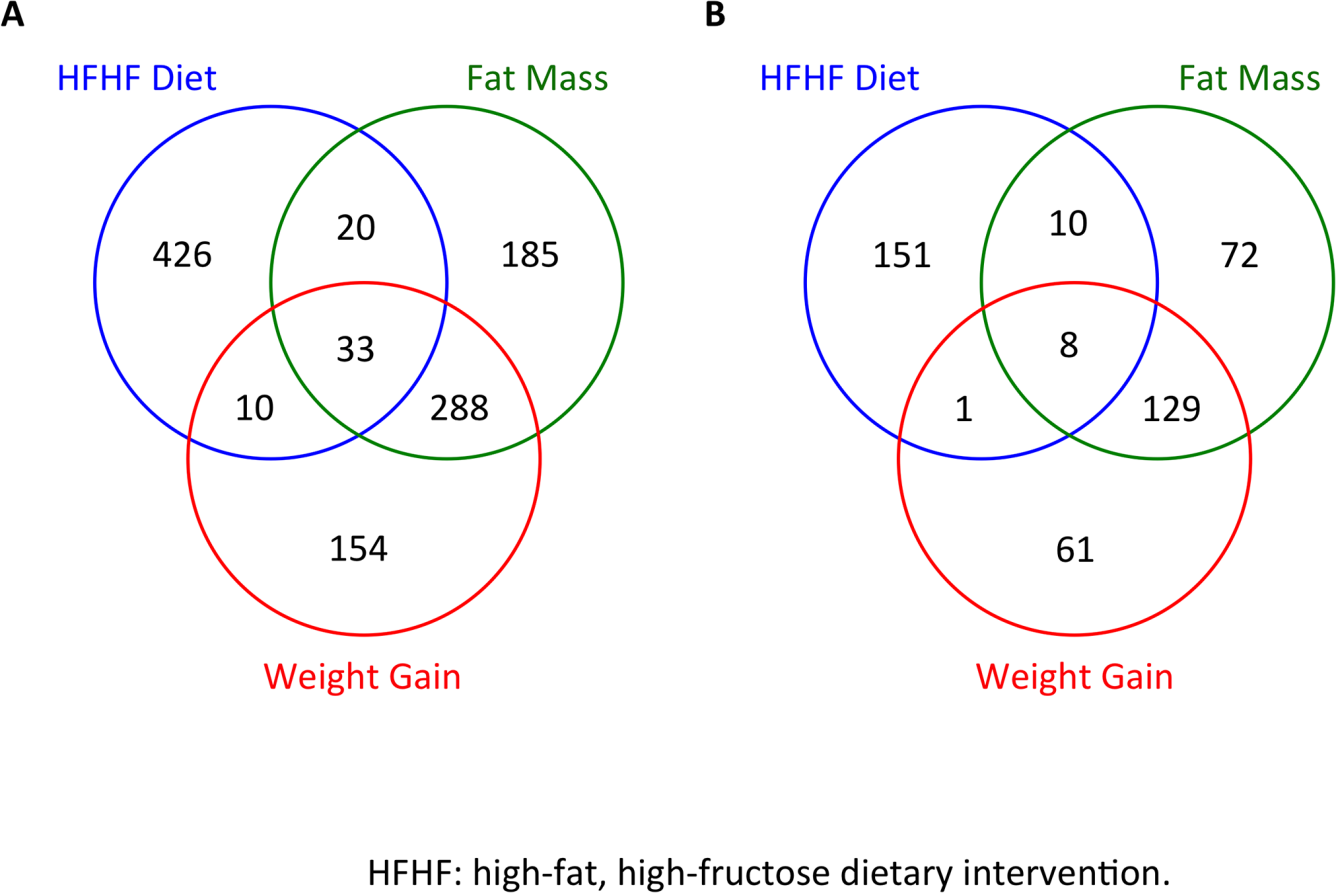
Venn Diagrams for Total Differentially Expressed Genes by Diet, Weight Gain and Fat Mass. A. all vervet mRNAs. B. all mRNAs that were annotated to human genes. (p,0.05, FDR<0.15).

Using RNA sequencing, 61.8 to 101.7 million total single-paired end reads per sample were received and 48.6 to 88.1 million reads were mappable to the draft vervet genome [18]. Approximately 1100 mRNA exhibited significant changes in response (p<0.05, FDR<0.15) to the HFHF diet within the CL or correlated with increases in body weight and/or fat mass (Fig 1A). Of these, 432 sequences were identified and annotated by homology to the human genome Fig 1B). Analysis of the transcriptome in each category (diet, weight gain and fat mass gain) identified subsets of differentially expressed genes (DEG). As expected, the majority of genes correlating with weight gain overlapped with those associated with increased fat mass and/or diet allocation. However, we also observed specific, mutually exclusive, subsets of genes responsive to dietary intervention, fat mass or weight gain only (S3 Table).

### Observed Changes in miRNA Gene Expression were Consistent with Development of Dysfunctional CL

Sequencing of the small RNA fraction identified 50 miRNAs, based upon homology to their human counterparts, of which 9 were differentially expressed (p<0.05, FDR<0.15) in response to HFHF diet (Table 3). These included members of the Let 7 family, miR-26a and miR-143, which are among most abundant miRNAs found in mouse, bovine, sheep and human ovaries [40–43]. Notably, several miRNAs induced in response to the HFHF diet were consistent with the development of dysfunctional CL. Specifically, Let-7b and miR -28 have been shown to inhibit progesterone and testosterone production in human granulosa cells (GC), while miR-26a and miR-28 suppress estrogen secretion [44–46]. Similarly, expression of let-7b, miR-26a, miR-28 and miR-143 were previously associated with decreased proliferation of GC, while let7b and miR-26a were found to promote GC apoptosis[45–47]. Additionally, we identified small nucleolar RNAs, splicing factors and several sequences, present in the vervet and other primate genomes which lack a human homolog; these may represent novel species specific miRs [48]. Several tRNA-derived fragments (tRFs) [49, 50], which are postulated to play a role in gene silencing mechanisms by interacting with canonical miR pathways [51, 52], also exhibited changes in abundance in response to the HFHF diet.

**Table 3:** Differentially Expressed Corpus Luteum miRNAs after High Fat High Fructose Diet.

miRNA	Fold Change	P
let7b-5p	56	0.002
let7e-5p	17,320	0.016
26a-5p	61,129	0.008
28-3p	4,381	0.008
143-5p	166,866	0.0001
186-5p	298	0.004
7193-5p	145,501	0.017
193b-3p	-15,448	0.017
486-5p	-25,555	0.015

### Integrated miRNA and mRNA Analysis

We used Ingenuity software to identify concordant changes in miRNAs and mRNAs. This approach evaluated an increase in any miRNA that was reflected by a corresponding reduction in its predicted target mRNA and *vice versa*. Among the 432 mRNAs differentially expressed in the vervet CL, changes in 52 were consistent with these criteria (Table 4). Among the 9 differentially expressed miRNAs, changes in 5 were consistent with corresponding changes in their mRNA targets. These miRNAs had 3 possible categorizations based on relevancy to luteal physiology and adiposity: (1) known links to both CL and adiposity; known links to (2) CL only or (3) adiposity only.

**Table 4:** Integrated Analysis of miRNAs and their mRNA Targets after High Fat High Fructose Diet.

MicroRNA (Fold Change)	Target mRNA Gene Symbol (Name)	Fold Change
hsa-miR-186-5p (298)	NAA38 (LSM8 homolog, U6 small nuclear RNA associated)	-29.0
	UBE2B (ubiquitin-conjugating enzyme E2B)	-22.0
	FAM204A (family with sequence similarity 204, member A)	-7.8
	EIF2S2 (eukaryotic translation initiation factor 2, subunit 2 beta)	-4.4
	MPC2 (mitochondrial pyruvate carrier 2)	-3.3
	ACTR10 (actin-related protein 10 homolog)	-2.8
	PDCD10 (programmed cell death 10)	-2.8
hsa-miR-193b-3p (-15,448)	SRSF6 (serine/arginine-rich splicing factor 6)	41.0
	TRIB2 (tribbles pseudokinase 2)	12.0
	UBP1 (upstream binding protein 1 (LBP-1a)	12.0
	TAOK2 (TAO kinase 2)	7.0
	SCARF1 (scavenger receptor class F, member 1)	6.0
	GABPA (GA binding protein transcription factor, α subunit) 60kDa)	5.0
	GPANK1(G patch domain and ankyrin repeats 1)	5.0
	PPM1F (protein phosphatase, Mg2+/Mn2+ dependent, 1F)	5.0
	STX16 (syntaxin 16)	5.0
	ABI2 (abl-interactor 2)	4.0
	CCNG2 (cyclin G2)	4.0
	FGF12 (fibroblast growth factor 12)	4.0
	CREBRF (CREB3 regulatory factor)	3.0
	FAM53C (family with sequence similarity 53, member C)	3.0
	RAPGEF5 (Rap guanine nucleotide exchange factor (GEF) 5)	3.0
	RGL1 (ral guanine nucleotide dissociation stimulator-like 1)	3.0
	ZBTB40 (zinc finger and BTB domain containing 40)	3.0
	DLG1 (discs, large homolog 1 (Drosophila)	2.0
	DYRK1A (dual-specificity TYR-(Y)-phos. regulated kinase 1A)	2.0
	ERAP2 (endoplasmic reticulum aminopeptidase 2)	2.0
	JAK2 (Janus kinase 2)	2.0
	SLC23A2 (solute carrier family 23,ascorbiate transporter, member 2	2.0
	ZNF562 (zinc finger protein 562)	2.0
hsa-miR-26a-5p (61,129)	MSMO1 (methylsterol monooxygenase 1)	-15.0
	VMA21 (VMA21 vacuolar H+-ATPase homolog)	-9.9
	NT5DC1 (5'-nucleotidase domain containing 1)	-9.0
	MAT2A (methionine adenosyltransferase II, alpha)	-6.0
	BCCIP (BRCA2 and CDKN1A interacting protein)	-4.9
	MTFMT (mitochondrial methionyl-tRNA. Fformyltransferase)	-4.6
	B4GALT4 (UDP-Gal:betaGlcNAc beta 1,4- GST, polypeptide 4	-3.4
	MCUR1 (mitochondrial calcium uniporter regulator 1)	-3.0
	PDCD10 (programmed cell death 10)	-2.8
	SRP19 (signal recognition particle 19kDa)	-2.6
hsa-miR-28-3p (4,831)	BCCIP (BRCA2 and CDKN1A interacting prtein) protein	-4.9
	PARL (presenilin associated, rhomboid-like)	-4.8
hsa-miR-486-5p (-25,555)	PTEN (phosphatase and tensin homolog)	6.0
	FLRT2 (fibronectin leucine rich transmembrane protein 2)	5.0
	ARHGAP5 (Rho GTPase activating protein 5)	4.0
	ZNF701 (zinc finger protein 701)	4.0
	GRAP (GRB2-related adaptor protein)	3.0
	TEK (TEK tyrosine kinase, endothelial)	3.0
	TTC31 (tetratricopeptide repeat domain 31)	3.0
	VPS37B (vacuolar protein sorting 37 homolog B)	3.0
	DYRK1A (dual-specificity TYR-(Y)-phos. regulated kinase 1A)	2.0
	PPP1R16A (protein phosphatase 1, regulatory subunit 16A)	2.0

Among the miRNAs that have been individually shown to be important for both CL function and adiposity, changes in miR-26 and miR-28 were notable. In addition to inhibition of sex steroid production in vitro by both of these miRNA [45], miR-28 is over expressed during the E2 drop following dominant follicle selection [53]. In our setting, both were up-regulated in response to HFHF diet, which is consistent with dysregulation of CL function. A notable down-regulated mRNA target of miR-28 was PARL, a critical regulator of mitochondrial morphology and function. Reduced PARL levels correlate with mitochondrial abnormalities in obesity and are linked with insulin resistance [54–56]. Maternal obesity and a high fat diet resulted in decreased expression of PARL in rats and it may play a role in metabolic programming [54]. Another down-regulated mRNA target of miR-28 was BCCIP, a cofactor for BRCA2, which functions as a progesterone-responsive gene involved in DNA repair and cell cycle control [57]. Among miR-26 targets, genes with the highest level of down-regulation were MSMO1 and VMA21. MSMO1 mediates LH stimulation of cholesterol biosynthesis [58, 59]. Deficiency in VMA21 results in impaired autophagy and endoplasmic reticulum stress [60, 61] and is associated with development of metabolic syndrome [62]. Taken together, our observed down-regulated mRNA targets of up-regulated miRNAs provide new mechanistic links between weight gain and CL function.

Among the miRNAs with known impact on CL function, only miR-186 was up-regulated. In follicular fluid, miR-186 increases in response to exogenous progesterone after ovulation [63]. *EIF2S2*, an mRNA target of miR-186 was down-regulated in this study. It has been implicated in differentiation of granulosa cells [64] and has been causally linked to a genetic variant of diminished ovarian reserve [65]. *EIF2S2* is a translation initiation factor that functions in the early steps of protein synthesis. It regulates angiogenesis via VEGF signaling due to accumulation of denatured proteins in stress and its dysfunction induces apoptosis of follicles [66]. Thus, down-regulation of *EIF2S2* implies decreased CL formation due to decreased angiogenesis.

Among the miRNA affected only in adiposity, miR-486 was down-regulated. MiR-486 has been shown to inhibit adipogenesis in human *in vitro* and animal *in vivo* obesity models [67, 68]. Thus, down-regulation of miR-486 may promote adipogenesis. In our setting, several of its up-regulated mRNA targets with known impact on CL function were detected. The target mRNA with the highest up-regulation was PTEN, a tumor suppressor and cell cycle regulator that inhibits CL granulosa cell differentiation and survival [69, 70]. Similarly, TEK/Tie2, an angiopoietin receptor, is implicated in CL angiogenesis and may mediate follicular atresia [71].

After HGHF diet, miR-193 was significantly down-regulated. It is down-regulated in adipose tissue from obese patients and is negatively correlated with BMI [72, 73]. Among its up-regulated mRNA targets, several were notable for known links with either CL function or diet-induced adiposity. Increased CREBRF (Luman recruiting factor) promotes apoptosis of granulosa cells [74]. *TRIB2* is known to promote visceral and ectopic fat accumulation [75, 76] and is implicated in dominant follicle selection [77]. Other up-regulated genes that are also targets of miR-193 included mediators of obesity, related inflammatory cytokine and leptin signaling (JAK2) [78, 79] as well as atherosclerosis (SCARF2/SREC-I) [80].

## Discussion

In this 10 month-long dietary intervention study, we have established a nonhuman primate (NHP) model to examine *de novo* weight gain in relation to luteal physiology. Due to their close phylogenetic relationship to humans, NHP are of special interest in modeling human disease. In studies of female reproduction, it is advantageous to use NHP models as they exhibit close similarities in the endocrine control of the menstrual cycle with regulatory mechanisms that are distinct from estrous species [81, 82]. Vervets are a small NHP species with a well-characterized menstrual cycle that mimics women in length and hormonal regulation [25–28]. Development of obesity in long-term vervet studies demonstrated an associated metabolic profile analogous to humans [30]. Our model allowed for invasive interventions, removal of the meticulously timed CL to collect tissue for analysis and paired design to maximize power in this costly setting. As ovulation was confirmed in all luteectomy cycles, this is the first study to uncover the response of the ovulating ovary to weight gain. Importantly, obesity is common in ovulatory women [8], leads to profound consequences on offspring health [10] and has not been adequately examined with respect to CL function. Thus, our work bridges a knowledge gap by creating a model of diet-induced weight gain and its effects on ovarian function. Use of a menstrual NHP species allowed us to apply invasive investigative tools that cannot be practically used in humans.

Monkeys consuming HFHF diet more than doubled their total body fat mass (106%). The average weight gain (20%) and the average percentage of body fat gain (66%) in the intervention group were comparable to other NHP high-fat feeding experiments [39, 83, 84]. While multiple NHP studies have examined the impact of adiposity on various organ systems, this is the first report of the ovary as the principal target organ, and represents the first comprehensive analysis of changes in expression of mRNAs and non-coding RNAs in the vervet CL in response to diet-induced weight gain.

Based upon homology to the human genome, we identified and jointly analyzed both mRNAs and 50 unique miRNAs that were expressed at detectable levels in the vervet CL. Specific changes in miRNA and mRNA expression profiles were consistent with impaired folliculogenesis and sex steroid function. Overall, HFHF diet-induced DEGs were consistent with a dysfunctional CL. We identified a multitude of genes highly relevant for luteal physiology, thus substantiating the choice of the model and approach used as profiling of vervet CL has not been published before. The DEGs included genes linked to premature ovarian failure (*NTRK2*)[85, 86], polycystic ovary syndrome (*MEF2A*)[87], signaling pathways regulating follicular development and angiogenesis, growth and survival of granulosa cells, and responses to gonadotropins (*BAK1*[88], *BMP1*[89], *BMP4*[89, 90], *GADD45A*[91], *IFNGR1*[92], *IGFBP1*[93], *JAK2*[94], *MAPILC3A*[95, 96], *NOS3*[97, 98], *NOTCH2*[99], *PLK2*[100, 101], *PTEN*[70], *SFRP4*[102], *VCAN*[103], *YY1*[104]). Further, transcripts implicated in steroidogenesis, cholesterol biosynthesis, transport and metabolism (AUP1[105], *FDFT1*[106], *PAWR*[107], *SLCO2B1*[108], *VAMP4*[109]), progesterone synthesis (*BLVRB*[110], BMP4[90]) and linked to obesity-related reduced fertility (*FANCC*[111], *PPT1*[112], *SFRP4*[102]) exhibited significant changes in expression (p<0.05, FDR<0.15). This underscores physiological relevance and utility of this model for further exploration in targeted and longer-term studies of high-fat feeding and its effects of hypothalamic-pituitary-ovarian axis, gestation and neonatal health.

Our CL miRNA data are novel with respect to the response to the intervention and joint analysis of target- miRNA interactions. While some of observed DEGs were previously reported in studies of either obesity or reproduction separately, we expand these data by the newly identified co-occurring links between the metabolic and reproductive axes.

Published reports suggest that let7b, miR-26a and miR-28 inhibit sex steroid secretion from GC, while let7b, miR-26a, miR-28 and miR-143 decrease GC proliferation and let7b and miR-26a promote GC apoptosis [46]. Providing mechanistic insight into impaired CL function with weight gain, several down regulated target genes were observed coincident with up-regulation of their predicted miRNA regulators. Several such genes represent our DEGs with the highest level of expression in response to HFHF and are known to regulate CL angiogenesis and differentiation, as well as cholesterol biosynthesis in response to LH, CL angiogenesis and differentiation. Specifically, down-regulated DEGs likely to regulate CL dysfunction include *MSMO1* (methylsterol monooxygenase located in endoplasmic reticulum membrane, involved in cholesterol biosynthesis and induced by LH in GCs [58, 59]), *VMA21* (vacuolar ATPase deficiency linked to autophagy and ER stress and metabolic syndrome [60–62]), and *PARL* (an integral mitochondrial protein that decreases mitochondrial abundance and integrity in response to insulin resistance [54–56]).

Conversely, *CREBRF* and *PTEN* were up-regulated and observed jointly with down-regulation of their predicted miRNA regulators, miR-193 and miR-486, respectively. *CREBRF* regulates apoptosis of GC cells [74], while ovary-specific deletion of *PTEN* causes premature activation of the primordial ovarian follicles [113], a process that is linked with diminished ovarian reserve and function and important for follicular atresia [114]. Other observed changes in DEGs were also consistent with systemic changes in metabolic regulation, mitochondrial dysfunction and ER stress, inflammation and lipotoxicity, which may underlie the reported decrease in ovarian function, steroid hormone secretion and concomitant reduction in fertility in obese women [115–118].

Limitations of our study included the fact that sequencing coverage was comprehensive but restricted by incomplete annotation of the vervet genome. Approximately 60% of potential mRNA sequences present in the draft vervet genome remain unannotated after human genome lift-over. Thus, we relied on genes with the highest homology to the human reference (i.e. the human lift-over) for both mRNA and miRNA analyses. While Next Generation sequencing of RNAs has been shown to be highly reproducible with little technical variation [119, 120], the limited amount of CL tissue obtained from the surgical procedures required the entire specimen to be used to provide sufficient materials for library preparation and sequencing analysis. Animal welfare and economic considerations did not allow procurement of additional independent samples for secondary analyses of protein or transcript levels. We initially hypothesized that weight gain due to HFHF diet would lead to decreased ovarian sex steroid secretion, as observed in obese ovulatory women [12, 16, 121]. However, our assessment of reproductive hormones did not reveal any significant changes after intervention, possibly due to inter-individual variation in hormone levels within each group and insufficient time of dietary intervention to develop overt reproductive endocrine dysfunction. Nonetheless, our observed phenotypic variation in response to dietary intervention is similar to that observed in humans and was unequivocally accompanied by significant weight, body fat and metabolic changes. Thus, we have established that our model represents an early, pre-clinical response of the ovarian transcriptome to an acute gain in adiposity.

In summary, we have demonstrated that a 10 month administration of an adipogenic diet results in doubling of fat mass, significant weight gain and substantial gene expression changes in the vervet CL. We report hitherto unreported links for genes and miRNAs regulating both luteal physiology and response to adiposity. The current report bridges a knowledge gap by creating a model of diet-induced weight gain and its effects on ovarian function in a menstrual NHP species with confirmed ovulation in each analyzed cycle. A critical shortcoming of using *in vivo* animal work to model women’s reproductive health is the long duration required for oocyte recruitment, CL timespan and gestation in women. Unlike rodents, vervets exhibit a well-characterized menstrual cycle with secretion of ovarian steroids and patterns of pituitary gonadotropins that mimic those of women. Vervets also develop obesity and associated metabolic profile similar to humans, while allowing invasive tissue acquisition for analysis. Using this model, we have documented for the first time changes in the CL transcriptome in response to diet-induced weight gain, which provide insight into the potential mechanisms mediating the CL dysfunction characteristic of obesity in humans. Alterations in miRNA expression that are implicated in regulation of folliculogenesis, sex steroid synthesis, and their corresponding target mRNA levels may underlie reduced fertility and adverse pregnancy outcomes linked to obesity. Future studies are well positioned to expand this model with full annotation of the whole vervet genome sequencing and mapping of more genes. The attractiveness of using this NHP model for reproductive studies is underscored by the phylogenetic link to people, close similarity of reproductive endocrine dynamics and the ability to apply powerful investigative tools that cannot practically be used in humans.

## Supporting Information

S1 Fig Study Design. (TIFF)

S1 File Supplementary Materials and Methods. (DOCX)

S2 File ARRIVE Guidelines Checklist. (PDF)

S1 Table Macronutrient Composition of Experimental Diets. (DOCX)

S2 Table Reproductive Characteristics. (DOCX)

S3 Table Dataset of Differentially Expressed mRNAs. (DOCX)

S4 Table mRNA Quality Control Report. (DOCX)

S5 Table miRNA Quality Control Report. (DOCX)
