# Supplementary figures and images for: Joint MiRNA/mRNA Expression Profiling Reveals Changes Consistent with Development of Dysfunctional Corpus Luteum after Weight Gain
Source: PLoS One. 2015 Aug 10;10(8):e0135163. doi: 10.1371/journal.pone.0135163 (PMC4530955; doi:10.1371/journal.pone.0135163)

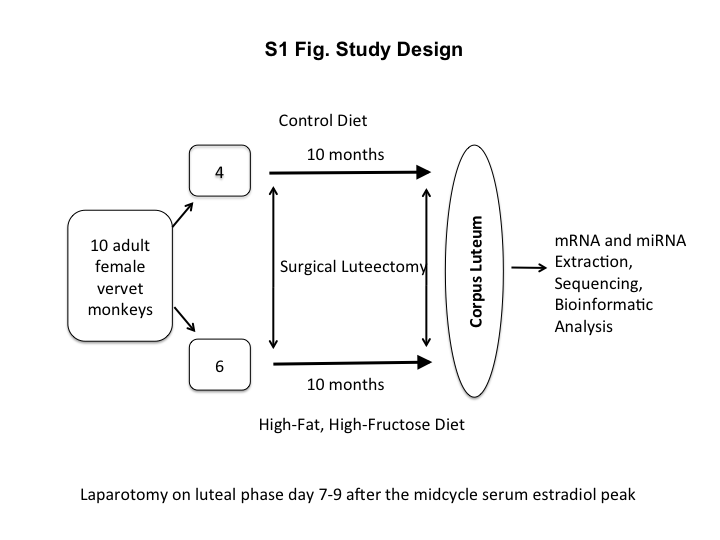

Supplement: S1 Fig — (TIFF) [file pone.0135163.s001.tiff]
