# Supplementary material for: Joint MiRNA/mRNA Expression Profiling Reveals Changes Consistent with Development of Dysfunctional Corpus Luteum after Weight Gain
Source: PLoS One. 2015 Aug 10;10(8):e0135163. doi: 10.1371/journal.pone.0135163 (PMC4530955; doi:10.1371/journal.pone.0135163)
Supplement: S1 File — (DOCX) [file pone.0135163.s002.docx]

**S1. Supplementary Materials and Methods**

**Animals Handling, Morphometrics and Urine collection**

Ten adult female vervet monkeys (*Chlorocebus aethiops sabaeus*) were selected randomly from the middle of distribution of body mass from the Vervet Research Colony at the Wake Forest Primate Center (WFRC, Winston-Salem, NC). All monkeys were within the normal weight range for this species and were sexually mature with a mean age of 6.8 years (Table 2). Monkeys were pair-housed at Wake Forest School of Medicine Primate Center / Center for Comparative Medicine and Research/ Friedberg Campus (Winston-Salem, NC). Monkeys were housed indoors in a climate controlled, temperature and humidity monitored room/building. The caging was USDA approved, steel Quad cages constructed of mesh flooring, removable dividers to allow horizontal movement between the two cages for each pair of monkeys, and a pan underneath to collect excrement and other waste. Monkeys were exposed to artificial lighting in a 12 hour, light-dark cycle from 6am to 6pm, with additional ambient light via windows in the hallway external to their housing room. Monkeys had ad-libitum access to water through water lixits and were fed 120kcal of experimental diet /kg of body weight once per day, as detailed in Dietary Intervention. In addition, feeding and foraging opportunities were provided 3-4 times per week (fresh fruits & vegetables, popcorn, sunflower seeds). For environmental enrichment, all cages were equipped with perches inside and hanging mirrors and puzzle feeders on the outside of the cage. All policies and procedures were done in compliance with state and federal laws, and regulations and guidelines established by the WFRC Animal Care and Use Committee. The WFRC Friedberg Campus is an Association for Assessment and Accreditation of Laboratory Animal Care AAALAC-accredited facility and all housing is AAALAC-and FDA approved.

External evidence of menstrual bleeding can be difficult to detect in vervets and therefore menses were determined using daily vaginal swabbing [1]. This technique that has been shown to be highly reliable over extended follow-up [2]. Monkeys were trained to present for vaginal swabbing using positive reinforcement with food rewards. Once trained, a cotton swab was inserted into the vagina to the level of the cervix, withdrawn and the presence of blood recorded. The first day of bleeding was recorded as day 1 of the menstrual cycle. Menses were recorded daily throughout the study.

Body weight, trunk length (distance from sternal notch to pubis) and waist circumference were measured monthly. Dual energy X-ray absorptiometry (DXA) was performed at baseline and after 10 months of dietary intervention. In house DXA (Hologic, Bedford, MA, USA) scanner was used for measurements of body composition. For each scan, monkeys were sedated with Ketamine HCL (15 mg/kg) intramuscularly, placed in the supine position and the tail was secured to the animal’s thigh to ensure that it was included in the scanning region. Scans were analyzed using the Hologic Software, APEX 3.3.

Urine was collected for 10 weeks at baseline and for 10 weeks after completion of the dietary intervention (3 to 5 day per week at baseline and daily after intervention). At 8 am every morning a mesh panel was inserted into each cage to separate pairs of monkeys and allow the collection of individual urine samples. Once the monkeys were separated, the metal pans underneath the mesh flooring were cleaned of all shavings and waste. Within 4 hours after cage preparation, a sterile 10 mL syringe was used to withdraw at least 5 mL of urine from the pan, avoiding collection of samples contaminated by water or feces. The urine was transferred to a tube for centrifugation at 10,000 rpm for 30 minutes and the supernatant was frozen.

**Power Analysis and Sample Size**

Findings from several independent groups indicate that influence of obesity on reproductive hormones does not appear to represent a threshold effect. Rather, reduction in reproductive hormones was observed with BMI in the overweight range (over 25) [3] and reduction in pregnancy rates was observed with BMI over 29 [4]. Our preliminary data accumulated in the study of women undergoing bariatric surgery suggest that obesity is associated with reduced serum LH and urinary progesterone metabolite, Pdg [5]. The basis for the power calculations is a defect in Pdg pulse amplitude of 78% (38.1 IU/liter vs. 181.3 IU/liter) in obese women vs. normal weight controls. Smaller differences result in a sample size as follows:

| N per group | Pdg difference,% | power | Two-tailed Alpha |
| --- | --- | --- | --- |
| 2 | 78 | 80 | 0.05 |
| 3 | 70 | 80 | 0.05 |
| 4 | 60 | 80 | 0.05 |
| 5 | 50 | 80 | 0.05 |
| 6 |  | 80 | 0.05 |

A sample size of 2 subjects should be sufficient if the between group differences are as large as they were in our preliminary human studies. However, the difference in body mass between the morbidly obese women and normal weight controls in our preliminary studies is likely larger than the weight difference expected at 10 months after the diet switch. In order to minimize the likelihood that only weak differences are noted at one-year point and there are not enough animals in the study to enable us to reach a statistically valid conclusion, we plan to study 4 monkeys per group to detect at least 60% difference in Pdg to examine this hypothesis thoroughly and will include 2 additional monkeys in eth experimental group to account for uninform adiposity acquisition. Thus, with power of 80% and a two-tailed alpha of 0.05, we used a sample size of 4 monkeys in the control group and 6 monkeys in the experimental group to detect a 60% difference in Pdg.

**Serum and Urine Analytes and Assays**

The urinary excretion of sex steroids closely corresponds to the serum concentrations of the parent hormones [6]. Urine was assayed for estrone conjugates (E1c) and pregnanediol glucuronide (Pdg) using previously described methods [7]. The E1c and Pdg levels were measured in duplicate by ELISA using antibodies and conjugate tracers provided by the laboratory of Drs. Bill Lasley and Nancy Gee (University of California Davis). Quality controls reading close to the assay ED50 were used for calculation of coefficients of variation (CV). Interassay CV for E1c was 16.8% and intraassay CV was 6.2%. Corresponding CVs for Pdg were 18.5% and 11.1%, respectively. Hormone concentrations were adjusted for glycerol and normalized to creatinine [8]. A validated algorithm [9] was used to asses luteal function by a three-step process. First, Pdg nadir (the lowest sample concentration) during the first 5 samples after menses was determined. Second, a sustained 3-fold rise from the nadir was defined as evidence of ovulation. Third, the day of a 60% drop in the E1c/ Pdg ratio in proximity to the Pdg peak was set as day 1 of the luteal phase. Serum anti-mullerian hormone (AMH) was measured with ultrasensitive ELISA (Ansh Labs, Webster, TX) with interassay and intraassay CVs less than 5%. Fasting serum lipid profile (including total cholesterol and triglyceride) were collected and measured at the WFPC using enzymatic methods published previously [10]. Total serum adiponectin was measured by ELISA (Mercodia, Winston-Salem, NC). Morphometric and hormonal data were analyzed with t test (paired for within the group comparison or unpaired for between the group comparisons) if normally distributed or Mann Whitney U test if skewed. All statistical tests used a two-tailed alpha of 0.05. Analyses were performed using STATA 11 (StataCorp LP, College Station, TX).

**Luteectomy**

In order to time the luteal phase, serum estradiol levels were measured daily from menstrual cycle day 7 during the luteectomy cycle. The first day of low serum estradiol (defined as values less than 100 pg/ml[11]) after the midcycle estradiol peak was denoted day 1 of the luteal phase [12]. Luteectomies were performed twice on all monkeys, at baseline and after dietary intervention. A laparotomy approach was used to collect CL tissue on luteal day 7-9. Prior to surgery, monkeys were pre-medicated with atropine and then sedated with ketamine (15 mg/kg IM). They were prepared aseptically for a midline abdominal incision, intubated and anesthetized with isoflurane gas. A ventral midline abdominal incision was made allowing visualization of both ovaries. The CL-containing ovary was stabilized with small forceps on the utero-ovarian ligament and the vascular pedicle. A small linear incision was made in the ovarian tunic overlying the CL, followed by blunt dissection and resection of the CL. The ovarian tunic was closed and the monkey was recovered. All animals recovered without complications. Immediately after dissection, the CL tissue was rinsed with sterile saline and place directly into a sterile vial containing RNAlater (Invitrogen, Life Technologies, Grand Island, NY) for future gene expression analysis. The vial was rotated 5-6 of times to ensure coverage of the tissue with RNAlater and then placed in a refrigerator at 4C for 24hrs prior to transfer to a freezer at -70C. A small piece of CL was fixed in 10% formalin and processed for confirmatory histological evaluation [13].

**Dietary Intervention**

At baseline, the monkeys were fed a commercial non-human primate diet (Purina Monkey Chow) once daily in the afternoon. After completion of the baseline procedures, monkeys were randomly assigned to either adipogenic HFHF diet (n=6), similar to that used for previous studies in baboons [14] and cynomolgus monkeys [15] or a control diet (Table 1). Both semi-purified solid “cake” diets were formulated in the WFPC Diet Laboratory. The diets were formulated to be identical with respect to ingredients used (S1) and were matched with respect to the amount of calories derived from protein and carbohydrates, cholesterol, fiber content and caloric density. The diets differed in the percentage of calories derived from fat (control ~26% vs. HFHF ~38%) and from simple sugars (control ~11% vs. HFHF~ 30%). In addition to the HFHF diet, monkeys in this group were given daily access to a Kool-Aid drink containing 15ml of high fructose corn syrup per 100 ml of water, providing ~150-250 additional calories per day. The dietary intervention was initiated in a staggered fashion to allow efficient completion of tissue collection.

**RNA isolation, Library Preparation, and Sequencing**

CL total RNA was extracted using TRIzol® method followed by purification using MirVana RNA Isolation Kit (Ambion). The RNA concentration and integrity were assessed Agilent Bioanalyzer. (Agilent Technol., Palo Alto, CA). Extracted RNA was forwarded to the University of Colorado Genomics and Microarray Core for library construction. For the mRNA libraries, a total of 200-500 ng of total RNA was used to prepare the Illumina HiSeq libraries according to manufacturer’s instructions for the TruSeq RNA kit. This kit first isolates mRNA from total RNA using polyA selection, then fragments and primes the mRNA for creation of double-stranded cDNA fragments. The fragments are amplified, size selected and purified for cluster generation. For miRNA libraries, 1000 ng of total RNA was used to prepare the Illumina HiSeq libraries according to manufacturer’s instructions for the TruSeq Small RNA kit. The small RNA kit’s 3 prime adapter is specifically modified to target microRNAs and other small RNAs that have a 3 prime hydroxyl group resulting from enzymatic cleavage by Dicer or other RNA processing enzymes. Final libraries were prepared via size selection on agarose gel to enrich for the smaller 21bp miRNA. The 20 mRNA libraries were independently barcoded, pooled, and sequenced across 5 lanes of a 2x100bp Illumina HiSeq2000 flowcell, whereas the 20 miRNA libraries were pooled and sequenced across 2 lanes of a 1x50bp flowcell.

**Computational Analysis and Quality Control**

**mRNA profiling**

All sequence reads were trimmed to an overall quality score of Q15 [16], and any sequences that were trimmed to less than 75bp were subsequently removed. The remaining sequences were mapped to the Vervet genome using genomic short-read nucleotide alignment program (GSNAP) [17]. From the mapped reads, the transcript assembly program Cufflinks (<http://cufflinks.cbcb.umd.edu/index.html>) was used to calculate the prevalence of transcripts from each known gene based on normalized read counts [18]. Differential mRNA expression (p<0.05, FDR<0.15) in response to diet and correlations to changes in body weight and percentage of fat mass by DXA were tested by ANOVA and linear regression, respectively, using custom-build scripts in the statistical software R [19]. For mRNA, 81.1-93.1% of high quality reads per sample were kept. Of those, 92.4-94.6% of reads per sample were mapped to human genome (S5, mRNA QC report).

**miRNA profiling**

As the miRNA sequence reads are shorter than the 50bp reads generated, the first step in quality assessment of the miRNA reads was to identify the reverse complement of the reverse sequencing adapter. Those reads where no adapter was found on the 3’-end of the read included a sequence insert that was longer than expected for a miRNA; thus these were removed from further analysis. After adapter identification and quality trimming as above, the sequences were further filtered for inserts that were 21-24bp in length, allowing for 3’-modifications. 22-24bp reads were then truncated on the 3’-end to a maximum length of 21bp, and trimmed to 20bp total by removing the first 5’ base. As no vervet monkey-specific miRNA databases exist, it was necessary to take an agnostic approach to find all small RNAs that might be differentially expressed. Thus, reads that passed quality filtering were analyzed with a custom Python script to identify unique sequences, and produce a normalized read count using DESeq normalization [20]. Next the expression of the anonymous unique sequence types was quantified and a similar ANOVA and regression approach as above was used to determine differential expression based upon the dietary treatment (control vs. HFHF). Unique sequences that were determined to be differentially expressed were subsequently BLASTed against the human genome (http://genome.ucsc.edu/) for identification. For miRNA, we received between 2.9 to 34.6 million total reads per sample. Of those, we kept 7.8-28.3% of high quality reads per sample as described above (S6, miRNA QC report).

**Genome Annotation**

Transcript sequencing output was mapped with the current vervet genome assembly (Chlorocebus_sabeus 1.0). We utilized the Vervet Genome Sequencing Project [21] in collaboration with the National Human Genome Research Institute and the Genome Institute, Washington University School of Medicine. The draft vervet genome assembly displays a degree of contiguity that is second only to human among primate genomes [22]. This high quality assembly is available via NCBI for public access (http://www.ncbi.nlm.nih.gov/assembly/GCA_000409795.1/). The preliminary gene annotation in vervet monkey was generated by alignments of Ensembl human proteins from Ensembl release 73. The preliminary annotation file includes alignments of human Ensembl translations, as well as alignments of 19 vervet monkey protein sequences, 88 vervet monkey cDNA sequences, and 33,798 EST sequences using methods previously described [23]. Of the 20,721 Ensembl human proteins, 20,126 aligned with a percent identity and coverage both greater than 50%. This preliminary gene annotation file, along with *ab initio* gene predictions and alignments of sequences from UniProt, UniGene and the ENA vertebrate RNA collection, are available at pre-Ensembl (http://pre.ensembl.org/Chlorocebus_sabaeus/Info/Index/).

**Target Gene Prediction and Integrated Analysis**

The selected miRNAs that were differentially expressed were further analyzed to identify the networks and pathways targets. Target prediction and integration of miRNA and mRNA bioinformatics analyses were done by Ingenuity Pathway Analysis (IPA) Software (Ingenuity Systems, Inc., Redwood City, California)). Highly predicted targets from Ingenuity Expert Findings were used. Ingenuity Pathway Analysis analyses the RNA expression data in the context of known biological response and regulatory networks, as well as other higher-order response pathways to assign functional information and biological relevance[24]. The DEG that corresponded as miRNA targets were exported to the Database for Annotation, Visualization and Integrated Discovery (DAVID) for enrichment of molecular functions [25].

**References**

1. Molskness TA, Hess DL, Maginnis GM, Wright JW, Fanton JW, et al. (2007) Characteristics and regulation of the ovarian cycle in vervet monkeys (Chlorocebus aethiops). American Journal of Primatology 69: 890-900.

2. Carroll RL, Mah K, Fanton JW, Maginnis GN, Brenner RM, et al. (2007) Assessment of menstruation in the vervet (Cercopithecus aethiops). American Journal of Primatology 69: 901-916.

3. Santoro N, Lasley B, McConnell D, Allsworth J, Crawford S, et al. (2004) Body size and ethnicity are associated with menstrual cycle alterations in women in the early menopausal transition: The Study of Women's Health across the Nation (SWAN) Daily Hormone Study. Journal of Clinical Endocrinology & Metabolism 89: 2622-2631.

4. van der Steeg JW, Steures P, Eijkemans MJ, Habbema JD, Hompes PG, et al. (2008) Obesity affects spontaneous pregnancy chances in subfertile, ovulatory women. Hum Reprod 23: 324-328.

5. Jain A, Polotsky AJ, Rochester D, Berga SL, Loucks T, et al. (2007) Pulsatile Luteinizing Hormone Amplitude and Progesterone Metabolite Excretion Are Reduced in Obese Women. Journal of Clinical Endocrinology & Metabolism 92: 2468-2473.

6. Munro C, Stabenfeldt G, Cragun J, Addiego L, Overstreet J, et al. (1991) Relationship of serum estradiol and progesterone concentrations to the excretion profiles of their major urinary metabolites as measured by enzyme immunoassay and radioimmunoassay. Clinical Chemistry 37: 838-844.

7. Kundu MC, May MC, Chosich J, Bradford AP, Lasley B, et al. (2013) Assessment of luteal function in the vervet monkey as a means to develop a model for obesity-related reproductive phenotype. Systems biology in reproductive medicine 59: 74-81.

8. Taussky HH (1954) A microcolorimetric determination of creatine in urine by the Jaffe reaction. Journal of Biological Chemistry 208: 853-862.

9. Santoro N, Brown JR, Adel T, Skurnick J (1996) Characterization of reproductive hormonal dynamics in the perimenopause. Journal of Clinical Endocrinology & Metabolism 81: 1495-1501.

10. Walker SE, Register TC, Appt SE, Adams MR, Clarkson TB, et al. (2008) Plasma lipid–dependent and–independent effects of dietary soy protein and social status on atherogenesis in premenopausal monkeys: implications for postmenopausal atherosclerosis burden. Menopause (New York, NY) 15: 950.

11. Bogan RL, Murphy MJ, Hennebold JD (2009) Dynamic changes in gene expression that occur during the period of spontaneous functional regression in the rhesus macaque corpus luteum. Endocrinology 150: 1521-1529.

12. Stephens SM, Pau FKY, Yalcinkaya TM, May MC, Berga SL, et al. (2013) Assessing the Pulsatility of Luteinizing Hormone in Female Vervet Monkeys (Chlorocebus aethiops sabaeus). Comparative medicine 63: 432-438.

13. Stephens SM, Pau FK, Yalcinkaya TM, May MC, Berga SL, et al. (2013) Assessing the pulsatility of luteinizing hormone in female vervet monkeys (Chlorocebus aethiops sabaeus). Comparative medicine 63: 432.

14. Higgins PB, Bastarrachea RA, Lopez-Alvarenga JC, Garcia-Forey M, Proffitt JM, et al. (2010) Eight week exposure to a high sugar high fat diet results in adiposity gain and alterations in metabolic biomarkers in baboons (Papio hamadryas sp.). Cardiovasc Diabetol 9: 71.

15. Mubiru JN, Garcia-Forey M, Higgins PB, Hemmat P, Cavazos NE, et al. (2011) A preliminary report on the feeding of cynomolgus monkeys (Macaca fascicularis) with a high-sugar high-fat diet for 33 weeks. Journal of Medical Primatology 40: 335-341.

16. Ewing B, Green P (1998) Base-calling of automated sequencer traces using phred. II. Error probabilities. Genome research 8: 186-194.

17. Wu TD, Nacu S (2010) Fast and SNP-tolerant detection of complex variants and splicing in short reads. Bioinformatics 26: 873-881.

18. Trapnell C, Hendrickson DG, Sauvageau M, Goff L, Rinn JL, et al. (2013) Differential analysis of gene regulation at transcript resolution with RNA-seq. Nat Biotechnol 31: 46-53.

19. R_Core_Team (2014) R: a language and environment for statistical computing (version 3.1. 0). Vienna: R Foundation for Statistical Computing. Available at <http://wwwR-projectorg>.

20. Anders S, Huber W (2010) Differential expression analysis for sequence count data. Genome biol 11: R106.

21. Jasinska AJ, Lin MK, Choi O-W, DeYoung J, Grujic O, et al. (2012) A non-human primate system for large-scale genetic studies of complex traits. Human molecular genetics 21: 3307-3316.

22. Jasinska AJ, Schmitt CA, Cantor RM, Dewar K, Jentsch JD, et al. (2013) Systems Biology of the Vervet Monkey. Institute of Laboratory Animal Resources 54: 122-143.

23. Flicek P, Amode MR, Barrell D, Beal K, Brent S, et al. (2012) Ensembl 2012. Nucleic acids research 40: D84-D90.

24. <http://www.ingenuity.com/>.

25. Dennis Jr G, Sherman BT, Hosack DA, Yang J, Gao W, et al. (2003) DAVID: database for annotation, visualization, and integrated discovery. Genome biol 4: P3.
