# Supplementary material for: Joint MiRNA/mRNA Expression Profiling Reveals Changes Consistent with Development of Dysfunctional Corpus Luteum after Weight Gain
Source: PLoS One. 2015 Aug 10;10(8):e0135163. doi: 10.1371/journal.pone.0135163 (PMC4530955; doi:10.1371/journal.pone.0135163)
Supplement: S1 Table — (DOCX) [file pone.0135163.s004.docx]

**S1 Table.** Macronutrient Composition of Experimental Diets^[[1]](#endnote-1)^

| **Composition** | **Control Diet** | **High-Fat, High-Fructose Diet** |
| --- | --- | --- |
|  |  |  |
| **Major sources of fats and cholesterol, g per 100g of diet** | | |
| Lard | 3 | 6 |
| Butter | 1.5 | 3 |
| Corn oil | 3 | 5 |
| Soybean oil | 1.5 | 2 |
| Crystalline cholesterol | 0.002 | 0.002 |
|  |  |  |
| **Major sources of**  c**arbohydrate , g per 100g of diet** | | |
| Starch (dextrin) | 11 | 3.6 |
| Wheat Flour | 40 | 34 |
| Cellulose Fiber | 12.6 | 10.3 |
| Sucrose | 3 | 3 |
| High Fructose corn syrup | 2.4 | 10 |
|  |  |  |

1. Both diets contained equal amounts of Vitamin Mix (Teklad TD85529 Vitamin Mix for Primates, Harlan laboratories, WI) and Mineral Mix (Teklad TD 93144 Mineral Mix, Harlan Laboratories, WI) and were supplemented with calcium carbonate and calcium phosphate to ensure a 1 to 1 ratio of calcium to phosphorous [↑](#endnote-ref-1)
