# Supplementary material for: Joint MiRNA/mRNA Expression Profiling Reveals Changes Consistent with Development of Dysfunctional Corpus Luteum after Weight Gain
Source: PLoS One. 2015 Aug 10;10(8):e0135163. doi: 10.1371/journal.pone.0135163 (PMC4530955; doi:10.1371/journal.pone.0135163)
Supplement: S2 Table — (DOCX) [file pone.0135163.s005.docx]

**S2 Table. Reproductive Characteristics ***

| **Variable** | **Control Diet**  **(n=4)** | **High Fat High Fructose Diet**  **(n=6)** |
| --- | --- | --- |
|  |  |  |
| Menstrual cycle length, days | 28.2 (0.8) ^0.34^  26.6 (1.0) ^0.19^ | 30.1 (0.5) ^0.34^  28.6 (1.1) ^0.21^ |
| AMH at baseline, ng/ml | 6.6 (1.6) | 4.0 (1.0) |
| Urinary Hormone Metabolites |  |  |
| Mean Luteal pdg, ng/mgCr |  |  |
| Baseline | 331.7 (65.1) ^0.34^ | 292.9 (75.0) ^0.34^ |
| 10 months | 398.7 (61.3) ^0.34^ | 386.6 (36.2) ^0.29^ |
| Mean Follicular E1c, ng/mgCr |  |  |
| Baseline | 105.2 (14.3) ^0.34^ | 97.1 (11.4) ^0.34^ |
| 10 months | 74.2 (6.9) ^0.22^ | 72.5 (2.3) ^0.12^ |
|  |  |  |

Values in table are mean (standard error of mean)

* Superscripts are P values for within the group comparisons for 10 month values (vs. baseline)
