# Supplementary material for: Joint MiRNA/mRNA Expression Profiling Reveals Changes Consistent with Development of Dysfunctional Corpus Luteum after Weight Gain
Source: PLoS One. 2015 Aug 10;10(8):e0135163. doi: 10.1371/journal.pone.0135163 (PMC4530955; doi:10.1371/journal.pone.0135163)
Supplement: S3 Table — (DOCX) [file pone.0135163.s006.docx]

| **S3 Table. Dataset of Differentially Expressed mRNAs** | | | | |
| --- | --- | --- | --- | --- |
|  | | | | |
|  |  | **Genes responsive exclusively to High Fat High Fructose Diet** |  |  |
| # | **Symbol** | **Entrez Gene Name** | **Fold Change** | **p-value** |
| 1 | ABI2 | abl-interactor 2 | 4.0 | 0.023 |
| 2 | ADAMTS5 | ADAM metallopeptidase with thrombospondin type 1 motif, 5 | 4.0 | 0.020 |
| 3 | ADIRF | adipogenesis regulatory factor | -9.1 | 0.024 |
| 4 | AFAP1L1 | actin filament associated protein 1-like 1 | 10.0 | 0.024 |
| 5 | AKAP8L | A kinase (PRKA) anchor protein 8-like | 12.0 | 0.015 |
| 6 | AKIP1 | A kinase (PRKA) interacting protein 1 | -3.5 | 0.037 |
| 7 | AKTIP | AKT interacting protein | -7.0 | 0.013 |
| 8 | AMZ2 | archaelysin family metallopeptidase 2 | -3.2 | 0.033 |
| 9 | ANKRD50 | ankyrin repeat domain 50 | 3.0 | 0.042 |
| 10 | ARID5B | AT rich interactive domain 5B (MRF1-like) | 5.0 | 0.003 |
| 11 | ARMCX6 | armadillo repeat containing, X-linked 6 | 2.0 | 0.015 |
| 12 | ATF7IP | activating transcription factor 7 interacting protein | 3.0 | 0.040 |
| 13 | ATG10 | autophagy related 10 | -3.0 | 0.045 |
| 14 | ATP6AP2 | ATPase, H+ transporting, lysosomal accessory protein 2 | -3.6 | 0.009 |
| 15 | ATP6V1E1 | ATPase, H+ transporting, lysosomal 31kDa, V1 subunit E1 | -7.3 | 0.047 |
| 16 | B4GALT4 | UDP-Gal:betaGlcNAc beta 1,4- galactosyltransferase, polypeptide 4 | -3.4 | 0.038 |
| 17 | BCCIP | BRCA2 and CDKN1A interacting protein | -4.9 | 0.045 |
| 18 | BLVRB | biliverdin reductase B (flavin reductase (NADPH)) | -8.0 | 0.036 |
| 19 | BMP1 | bone morphogenetic protein 1 | 11.0 | 0.042 |
| 20 | BRD8 | bromodomain containing 8 | 4.0 | 0.032 |
| 21 | BTBD9 | BTB (POZ) domain containing 9 | 2.0 | 0.044 |
| 22 | C4orf27 | chromosome 4 open reading frame 27 | -10.5 | 0.021 |
| 23 | C9orf9 | chromosome 9 open reading frame 9 | -25.6 | 0.031 |
| 24 | CBWD2 | COBW domain containing 2 | -26.5 | 0.033 |
| 25 | CCDC109B | coiled-coil domain containing 109B | -9.1 | 0.023 |
| 26 | CCDC23 | coiled-coil domain containing 23 | -20.0 | 0.040 |
| 27 | CCNG2 | cyclin G2 | 4.0 | 0.035 |
| 28 | CCNL1 | cyclin L1 | 5.0 | 0.043 |
| 29 | CGGBP1 | CGG triplet repeat binding protein 1 | 11.0 | 0.049 |
| 30 | CHMP4A | charged multivesicular body protein 4A | -4.0 | 0.048 |
| 31 | CHPT1 | choline phosphotransferase 1 | -4.0 | 0.005 |
| 32 | CLEC3B | C-type lectin domain family 3, member B | 10.0 | 0.004 |
| 33 | CNST | consortin, connexin sorting protein | 2.0 | 0.038 |
| 34 | COL12A1 | collagen, type XII, alpha 1 | 4.0 | 0.050 |
| 35 | CTNNBIP1 | catenin, beta interacting protein 1 | 3.0 | 0.021 |
| 36 | CYYR1 | cysteine/tyrosine-rich 1 | 8.0 | 0.027 |
| 37 | D2HGDH | D-2-hydroxyglutarate dehydrogenase | 14.0 | 0.011 |
| 38 | DDX47 | DEAD (Asp-Glu-Ala-Asp) box polypeptide 47 | -3.0 | 0.001 |
| 39 | DENR | density-regulated protein | -3.4 | 0.021 |
| 40 | DERA | deoxyribose-phosphate aldolase (putative) | -5.1 | 0.015 |
| 41 | DHRS7 | dehydrogenase/reductase (SDR family) member 7 | -15.0 | 0.004 |
| 42 | DNAJC2 | DnaJ (Hsp40) homolog, subfamily C, member 2 | -4.0 | 0.022 |
| 43 | DUSP11 | dual specificity phosphatase 11 (RNA/RNP complex 1-interacting) | 6.0 | 0.016 |
| 44 | ETF1 | eukaryotic translation termination factor 1 | -8.5 | 0.034 |
| 45 | ETV3 | ets variant 3 | 4.0 | 0.043 |
| 46 | EXOC3L1 | exocyst complex component 3-like 1 | 8.0 | 0.042 |
| 47 | EXOSC9 | exosome component 9 | -3.0 | 0.008 |
| 48 | FAM177A1 | family with sequence similarity 177, member A1 | -3.6 | 0.002 |
| 49 | FANCC | Fanconi anemia, complementation group C | 3.0 | 0.040 |
| 50 | FEZ2 | fasciculation and elongation protein zeta 2 (zygin II) | -45.4 | 0.007 |
| 51 | GALNT10 | polypeptide N-acetylgalactosaminyltransferase 10 | 3.0 | 0.039 |
| 52 | GNL2 | guanine nucleotide binding protein-like 2 (nucleolar) | -6.0 | 0.009 |
| 53 | GON4L | gon-4-like (C. elegans) | 2.0 | 0.017 |
| 54 | GOPC | golgi-associated PDZ and coiled-coil motif containing | 4.0 | 0.025 |
| 55 | GPN3 | GPN-loop GTPase 3 | -7.1 | 0.016 |
| 56 | GRAP | GRB2-related adaptor protein | 3.0 | 0.050 |
| 57 | IFNGR1 | interferon gamma receptor 1 | -13.0 | 0.039 |
| 58 | IFT52 | intraflagellar transport 52 homolog (Chlamydomonas) | -7.5 | 0.047 |
| 59 | ITPR3 | inositol 1,4,5-trisphosphate receptor, type 3 | 3.0 | 0.037 |
| 60 | JAK2 | Janus kinase 2 | 2.0 | 0.006 |
| 61 | KANK3 | KN motif and ankyrin repeat domains 3 | 5.0 | 0.039 |
| 62 | KIAA0040 | KIAA0040 | 3.0 | 0.042 |
| 63 | KIF7 | kinesin family member 7 | 3.0 | 0.020 |
| 64 | LIPA | lipase A, lysosomal acid, cholesterol esterase | -7.1 | 0.025 |
| 65 | LRRC24 | leucine rich repeat containing 24 | 4.0 | 0.010 |
| 66 | LRRC28 | leucine rich repeat containing 28 | -12.1 | 0.046 |
| 67 | LRRC37A3 (includes others) | leucine rich repeat containing 37, member A3 | 5.0 | 0.029 |
| 68 | MACF1 | microtubule-actin crosslinking factor 1 | 4.0 | 0.033 |
| 69 | MAP1LC3A | microtubule-associated protein 1 light chain 3 alpha | 11.0 | 0.045 |
| 70 | MAPK11 | mitogen-activated protein kinase 11 | 3.0 | 0.017 |
| 71 | MAT2A | methionine adenosyltransferase II, alpha | -6.0 | 0.028 |
| 72 | MCUR1 | mitochondrial calcium uniporter regulator 1 | -3.0 | 0.029 |
| 73 | MDM2 | MDM2 oncogene, E3 ubiquitin protein ligase | 4.0 | 0.050 |
| 74 | MED13L | mediator complex subunit 13-like | 2.0 | 0.045 |
| 75 | METTL16 | methyltransferase like 16 | 2.0 | 0.050 |
| 76 | METTL17 | methyltransferase like 17 | -2.0 | 0.014 |
| 77 | MMADHC | methylmalonic aciduria (cobalamin deficiency) cblD type, with homocystinuria | -4.2 | 0.048 |
| 78 | MPHOSPH10 | M-phase phosphoprotein 10 (U3 small nucleolar ribonucleoprotein) | -3.0 | 0.015 |
| 79 | MRPL10 | mitochondrial ribosomal protein L10 | -5.0 | 0.016 |
| 80 | MTFMT | mitochondrial methionyl-tRNA formyltransferase | -4.6 | 0.029 |
| 81 | NAAA | N-acylethanolamine acid amidase | -2.0 | 0.028 |
| 82 | NAE1 | NEDD8 activating enzyme E1 subunit 1 | -10.6 | 0.003 |
| 83 | NDRG3 | NDRG family member 3 | 8.0 | 0.028 |
| 84 | NDUFB10 | NADH dehydrogenase (ubiquinone) 1 beta subcomplex, 10, 22kDa | -8.0 | 0.015 |
| 85 | NEK9 | NIMA-related kinase 9 | 10.0 | 0.016 |
| 86 | NFKBIA | nuclear factor of kappa light polypeptide gene enhancer in B-cells inhibitor, alpha | 8.0 | 0.036 |
| 87 | NFU1 | NFU1 iron-sulfur cluster scaffold homolog (S. cerevisiae) | -3.3 | 0.042 |
| 88 | NOS3 | nitric oxide synthase 3 (endothelial cell) | 4.0 | 0.027 |
| 89 | NPDC1 | neural proliferation, differentiation and control, 1 | 14.0 | 0.039 |
| 90 | NRIP2 | nuclear receptor interacting protein 2 | 2.0 | 0.033 |
| 91 | NTPCR | nucleoside-triphosphatase, cancer-related | 4.0 | 0.033 |
| 92 | PAIP1 | poly(A) binding protein interacting protein 1 | -3.2 | 0.039 |
| 93 | PAN2 | PAN2 poly(A) specific ribonuclease subunit | 4.0 | 0.024 |
| 94 | PAPSS1 | 3'-phosphoadenosine 5'-phosphosulfate synthase 1 | -200.2 | 0.008 |
| 95 | PARL | presenilin associated, rhomboid-like | -4.8 | 0.036 |
| 96 | PARP2 | poly (ADP-ribose) polymerase 2 | -2.0 | 0.014 |
| 97 | PCNT | pericentrin | 2.0 | 0.029 |
| 98 | PELI1 | pellino E3 ubiquitin protein ligase 1 | 2.0 | 0.026 |
| 99 | PHF20 | PHD finger protein 20 | 2.0 | 0.018 |
| 100 | PIEZO2 | piezo-type mechanosensitive ion channel component 2 | 3.0 | 0.043 |
| 101 | PLEKHA1 | pleckstrin homology domain containing, family A (phosphoinositide binding specific) member 1 | 3.0 | 0.047 |
| 102 | PLK2 | polo-like kinase 2 | 16.0 | 0.038 |
| 103 | POLR2D | polymerase (RNA) II (DNA directed) polypeptide D | -10.0 | 0.003 |
| 104 | POLR2L | polymerase (RNA) II (DNA directed) polypeptide L, 7.6kDa | -21.0 | 0.026 |
| 105 | PPIH | peptidylprolyl isomerase H (cyclophilin H) | -7.0 | 0.001 |
| 106 | PPP1R8 | protein phosphatase 1, regulatory subunit 8 | -3.0 | 0.011 |
| 107 | PPP6C | protein phosphatase 6, catalytic subunit | -4.0 | 0.014 |
| 108 | PTEN | phosphatase and tensin homolog | 6.0 | 0.002 |
| 109 | RAPGEF2 | Rap guanine nucleotide exchange factor (GEF) 2 | 3.0 | 0.031 |
| 110 | RAPGEF5 | Rap guanine nucleotide exchange factor (GEF) 5 | 3.0 | 0.036 |
| 111 | RARRES1 | retinoic acid receptor responder (tazarotene induced) 1 | -859.9 | 0.038 |
| 112 | RBM26 | RNA binding motif protein 26 | 2.0 | 0.035 |
| 113 | RGP1 | RGP1 retrograde golgi transport homolog (S. cerevisiae) | 5.0 | 0.024 |
| 114 | SAAL1 | serum amyloid A-like 1 | -4.7 | 0.044 |
| 115 | SCARF1 | scavenger receptor class F, member 1 | 6.0 | 0.044 |
| 116 | SERPINB6 | serpin peptidase inhibitor, clade B (ovalbumin), member 6 | -3.9 | 0.012 |
| 117 | SLC25A26 | solute carrier family 25 (S-adenosylmethionine carrier), member 26 | -4.0 | 0.001 |
| 118 | SLC30A5 | solute carrier family 30 (zinc transporter), member 5 | -2.0 | 0.028 |
| 119 | SLC35D2 | solute carrier family 35 (UDP-GlcNAc/UDP-glucose transporter), member D2 | -6.9 | 0.024 |
| 120 | SMARCE1 | SWI/SNF related, matrix associated, actin dependent regulator of chromatin, subfamily e, member 1 | -7.0 | 0.007 |
| 121 | SOS1 | son of sevenless homolog 1 (Drosophila) | 2.0 | 0.026 |
| 122 | SPRED1 | sprouty-related, EVH1 domain containing 1 | 7.0 | 0.044 |
| 123 | SRSF6 | serine/arginine-rich splicing factor 6 | 41.0 | 0.046 |
| 124 | STX16 | syntaxin 16 | 5.0 | 0.044 |
| 125 | SUGP2 | SURP and G patch domain containing 2 | 6.0 | 0.037 |
| 126 | TBKBP1 | TBK1 binding protein 1 | 3.0 | 0.030 |
| 127 | TEK | TEK tyrosine kinase, endothelial | 3.0 | 0.036 |
| 128 | THOC7 | THO complex 7 homolog (Drosophila) | -5.7 | 0.043 |
| 129 | TMEM120B | transmembrane protein 120B | 4.0 | 0.025 |
| 130 | TMEM53 | transmembrane protein 53 | -2.0 | 0.024 |
| 131 | TRAPPC3 | trafficking protein particle complex 3 | -14.0 | 0.001 |
| 132 | TRIM32 | tripartite motif containing 32 | 3.0 | 0.025 |
| 133 | TRNAU1AP | tRNA selenocysteine 1 associated protein 1 | -7.0 | 0.012 |
| 134 | TTC31 | tetratricopeptide repeat domain 31 | 3.0 | 0.016 |
| 135 | TXNL1 | thioredoxin-like 1 | -3.9 | 0.031 |
| 136 | UPF2 | UPF2 regulator of nonsense transcripts homolog (yeast) | 2.0 | 0.043 |
| 137 | VMA21 | VMA21 vacuolar H+-ATPase homolog (S. cerevisiae) | -9.9 | 0.011 |
| 138 | WDR41 | WD repeat domain 41 | -4.4 | 0.044 |
| 139 | ZBTB40 | zinc finger and BTB domain containing 40 | 3.0 | 0.011 |
| 140 | ZC3H13 | zinc finger CCCH-type containing 13 | 4.0 | 0.021 |
| 141 | ZEB2 | zinc finger E-box binding homeobox 2 | 6.0 | 0.017 |
| 142 | ZNF318 | zinc finger protein 318 | 2.0 | 0.030 |
| 143 | ZNF444 | zinc finger protein 444 | 3.0 | 0.019 |
| 144 | ZNF561 | zinc finger protein 561 | 2.0 | 0.048 |
| 145 | ZNF562 | zinc finger protein 562 | 2.0 | 0.026 |
| 146 | ZNF608 | zinc finger protein 608 | 3.0 | 0.034 |
| 147 | ZNF692 | zinc finger protein 692 | 4.0 | 0.038 |
| 148 | ZRANB1 | zinc finger, RAN-binding domain containing 1 | 3.0 | 0.013 |
| 149 | ZSCAN29 | zinc finger and SCAN domain containing 29 | 2.0 | 0.034 |
| 150 | ZSWIM6 | zinc finger, SWIM-type containing 6 | 2.0 | 0.027 |
| 151 | ZSWIM8 | zinc finger, SWIM-type containing 8 | 9.0 | 0.046 |
|  |  |  |  |  |
|  |  |  |  |  |
|  |  | **Genes responsive exclusively to Increased Fat Mass** |  |  |
|  | **Symbol** | **Entrez Gene Name** | **Log Ratio** | **p-value** |
|  |  |  |  |  |
| 1 | ARMC6 | armadillo repeat containing 6 | 0.035 | 0.042 |
| 2 | AUTS2 | autism susceptibility candidate 2 | 0.032 | 0.023 |
| 3 | BAK1 | BCL2-antagonist/killer 1 | 0.048 | 0.047 |
| 4 | BAP1 | BRCA1 associated protein-1 (ubiquitin carboxy-terminal hydrolase) | 0.061 | 0.046 |
| 5 | BICC1 | BicC family RNA binding protein 1 | 0.034 | 0.006 |
| 6 | BMP4 | bone morphogenetic protein 4 | 0.045 | 0.038 |
| 7 | BOP1 | block of proliferation 1 | 0.063 | 0.009 |
| 8 | BRIX1 | BRX1, biogenesis of ribosomes, homolog (S. cerevisiae) | -0.066 | 0.050 |
| 9 | C11orf84 | chromosome 11 open reading frame 84 | 0.039 | 0.044 |
| 10 | CBX6 | chromobox homolog 6 | 0.041 | 0.040 |
| 11 | CCNDBP1 | cyclin D-type binding-protein 1 | -0.057 | 0.022 |
| 12 | CDC42EP2 | CDC42 effector protein (Rho GTPase binding) 2 | 0.045 | 0.037 |
| 13 | CDR2L | cerebellar degeneration-related protein 2-like | 0.044 | 0.047 |
| 14 | COL5A2 | collagen, type V, alpha 2 | 0.344 | 0.029 |
| 15 | COL8A1 | collagen, type VIII, alpha 1 | 0.073 | 0.026 |
| 16 | CSNK1G2 | casein kinase 1, gamma 2 | 0.081 | 0.049 |
| 17 | DBN1 | drebrin 1 | 0.125 | 0.032 |
| 18 | DEF8 | differentially expressed in FDCP 8 homolog (mouse) | 0.025 | 0.031 |
| 19 | DFFA | DNA fragmentation factor, 45kDa, alpha polypeptide | 0.038 | 0.027 |
| 20 | DNTTIP2 | deoxynucleotidyltransferase, terminal, interacting protein 2 | -0.058 | 0.049 |
| 21 | FAM109B | family with sequence similarity 109, member B | 0.024 | 0.037 |
| 22 | FDFT1 | farnesyl-diphosphate farnesyltransferase 1 | -0.042 | 0.024 |
| 23 | GADD45A | growth arrest and DNA-damage-inducible, alpha | 0.072 | 0.030 |
| 24 | GEMIN7 | gem (nuclear organelle) associated protein 7 | 0.036 | 0.049 |
| 25 | GINS4 | GINS complex subunit 4 (Sld5 homolog) | 0.033 | 0.011 |
| 26 | GLMN | glomulin, FKBP associated protein | -0.016 | 0.047 |
| 27 | IFIT5 | interferon-induced protein with tetratricopeptide repeats 5 | -0.059 | 0.046 |
| 28 | INE1 | inactivation escape 1 (non-protein coding) | 0.152 | 0.019 |
| 29 | JDP2 | Jun dimerization protein 2 | 0.052 | 0.042 |
| 30 | KCTD12 | potassium channel tetramerization domain containing 12 | 0.044 | 0.017 |
| 31 | LATS2 | large tumor suppressor kinase 2 | 0.026 | 0.040 |
| 32 | LUZP1 | leucine zipper protein 1 | 0.03 | 0.043 |
| 33 | MBOAT1 | membrane bound O-acyltransferase domain containing 1 | 0.024 | 0.016 |
| 34 | MCEE | methylmalonyl CoA epimerase | -0.068 | 0.032 |
| 35 | MCM7 | minichromosome maintenance complex component 7 | 0.03 | 0.047 |
| 36 | MEF2A | myocyte enhancer factor 2A | 0.035 | 0.048 |
| 37 | MXD4 | MAX dimerization protein 4 | 0.18 | 0.040 |
| 38 | NOD1 | nucleotide-binding oligomerization domain containing 1 | 0.018 | 0.019 |
| 39 | NOTCH2 | notch 2 | 0.048 | 0.035 |
| 40 | NTRK2 | neurotrophic tyrosine kinase, receptor, type 2 | 0.056 | 0.024 |
| 41 | NTS | neurotensin | 0.212 | 0.021 |
| 42 | PEX6 | peroxisomal biogenesis factor 6 | 0.043 | 0.049 |
| 43 | PHF11 | PHD finger protein 11 | -0.039 | 0.033 |
| 44 | PIGP | phosphatidylinositol glycan anchor biosynthesis, class P | -0.087 | 0.030 |
| 45 | PRPF18 | pre-mRNA processing factor 18 | -0.038 | 0.027 |
| 46 | QARS | glutaminyl-tRNA synthetase | 0.074 | 0.026 |
| 47 | R3HCC1 | R3H domain and coiled-coil containing 1 | 0.049 | 0.030 |
| 48 | RAD17 | RAD17 homolog (S. pombe) | -0.017 | 0.040 |
| 49 | RYBP | RING1 and YY1 binding protein | 0.029 | 0.042 |
| 50 | SGPL1 | sphingosine-1-phosphate lyase 1 | 0.036 | 0.022 |
| 51 | SHC1 | SHC (Src homology 2 domain containing) transforming protein 1 | 0.258 | 0.033 |
| 52 | SIRT6 | sirtuin 6 | 0.018 | 0.044 |
| 53 | SLC37A4 | solute carrier family 37 (glucose-6-phosphate transporter), member 4 | 0.03 | 0.041 |
| 54 | SLCO2B1 | solute carrier organic anion transporter family, member 2B1 | 0.105 | 0.050 |
| 55 | SOX12 | SRY (sex determining region Y)-box 12 | 0.028 | 0.028 |
| 56 | SPIN2A/SPIN2B | spindlin family, member 2B | -0.013 | 0.042 |
| 57 | SPPL2A | signal peptide peptidase like 2A | -0.097 | 0.045 |
| 58 | SRI | sorcin | 0.047 | 0.023 |
| 59 | SRSF2 | serine/arginine-rich splicing factor 2 | 0.154 | 0.017 |
| 60 | TDRD3 | tudor domain containing 3 | -0.017 | 0.011 |
| 61 | TIFA | TRAF-interacting protein with forkhead-associated domain | -0.062 | 0.037 |
| 62 | TJAP1 | tight junction associated protein 1 (peripheral) | 0.038 | 0.028 |
| 63 | TMEM126A | transmembrane protein 126A | -0.084 | 0.040 |
| 64 | TMEM176B | transmembrane protein 176B | 0.396 | 0.030 |
| 65 | TOM1 | target of myb1 (chicken) | 0.059 | 0.024 |
| 66 | UBLCP1 | ubiquitin-like domain containing CTD phosphatase 1 | -0.026 | 0.037 |
| 67 | UNC50 | unc-50 homolog (C. elegans) | -0.127 | 0.028 |
| 68 | USF1 | upstream transcription factor 1 | 0.044 | 0.033 |
| 69 | WASL | Wiskott-Aldrich syndrome-like | 0.118 | 0.039 |
| 70 | YBEY | ybeY metallopeptidase (putative) | -0.036 | 0.044 |
| 71 | ZBTB17 | zinc finger and BTB domain containing 17 | 0.035 | 0.033 |
| 72 | ZNF787 | zinc finger protein 787 | 0.028 | 0.046 |
|  |  |  |  |  |
|  |  |  |  |  |
|  |  |  |  |  |
|  |  |  |  |  |
|  |  |  |  |  |
|  |  | **Genes responsive exclusively to Weight Gain** |  |  |
|  | **Symbol** | **Entrez Gene Name** | **Log Ratio** | **p-value** |
|  |  |  |  |  |
| 1 | ALKBH2 | alkB, alkylation repair homolog 2 (E. coli) | 0.063 | 0.045 |
| 2 | ALPK1 | alpha-kinase 1 | -0.061 | 0.037 |
| 3 | ARMCX1 | armadillo repeat containing, X-linked 1 | 0.092 | 0.039 |
| 4 | AUP1 | ancient ubiquitous protein 1 | 0.213 | 0.029 |
| 5 | BAMBI | BMP and activin membrane-bound inhibitor | 0.269 | 0.036 |
| 6 | C12orf10 | chromosome 12 open reading frame 10 | 0.189 | 0.041 |
| 7 | C1orf122 | chromosome 1 open reading frame 122 | 0.703 | 0.022 |
| 8 | C1QTNF5 | C1q and tumor necrosis factor related protein 5 | 0.354 | 0.017 |
| 9 | CARHSP1 | calcium regulated heat stable protein 1, 24kDa | 0.415 | 0.032 |
| 10 | CCBL1 | cysteine conjugate-beta lyase, cytoplasmic | 0.057 | 0.018 |
| 11 | CCDC85B | coiled-coil domain containing 85B | 0.751 | 0.012 |
| 12 | CDC37 | cell division cycle 37 | 0.425 | 0.045 |
| 13 | CDC42EP5 | CDC42 effector protein (Rho GTPase binding) 5 | 0.122 | 0.031 |
| 14 | CDPF1 | cysteine-rich, DPF motif domain containing 1 | 0.09 | 0.036 |
| 15 | CLDN5 | claudin 5 | 0.342 | 0.028 |
| 16 | CRELD2 | cysteine-rich with EGF-like domains 2 | 0.199 | 0.050 |
| 17 | CUTA | cutA divalent cation tolerance homolog (E. coli) | 0.111 | 0.037 |
| 18 | DRG2 | developmentally regulated GTP binding protein 2 | 0.12 | 0.031 |
| 19 | DUSP14 | dual specificity phosphatase 14 | 0.131 | 0.023 |
| 20 | DUSP7 | dual specificity phosphatase 7 | 0.098 | 0.027 |
| 21 | EGLN2 | egl-9 family hypoxia-inducible factor 2 | 0.351 | 0.048 |
| 22 | F2RL1 | coagulation factor II (thrombin) receptor-like 1 | 0.422 | 0.036 |
| 23 | FAM20A | family with sequence similarity 20, member A | 0.114 | 0.042 |
| 24 | GALK1 | galactokinase 1 | 0.111 | 0.014 |
| 25 | GINM1 | glycoprotein integral membrane 1 | -0.228 | 0.039 |
| 26 | GMPPA | GDP-mannose pyrophosphorylase A | 0.146 | 0.033 |
| 27 | GTF2H4 | general transcription factor IIH, polypeptide 4, 52kDa | 0.071 | 0.039 |
| 28 | HABP4 | hyaluronan binding protein 4 | 0.157 | 0.031 |
| 29 | LEMD2 | LEM domain containing 2 | 0.123 | 0.050 |
| 30 | LOC102724594/U2AF1 | U2 small nuclear RNA auxiliary factor 1 | 0.231 | 0.040 |
| 31 | MIER2 | mesoderm induction early response 1, family member 2 | 0.086 | 0.035 |
| 32 | MRPL9 | mitochondrial ribosomal protein L9 | 0.22 | 0.034 |
| 33 | MXRA7 | matrix-remodelling associated 7 | 0.647 | 0.045 |
| 34 | MYL6B | myosin, light chain 6B, alkali, smooth muscle and non-muscle | 0.318 | 0.045 |
| 35 | NME3 | NME/NM23 nucleoside diphosphate kinase 3 | 0.272 | 0.042 |
| 36 | NPM3 | nucleophosmin/nucleoplasmin 3 | 0.4 | 0.014 |
| 37 | NPRL2 | nitrogen permease regulator-like 2 (S. cerevisiae) | 0.063 | 0.039 |
| 38 | NTMT1 | N-terminal Xaa-Pro-Lys N-methyltransferase 1 | 0.142 | 0.044 |
| 39 | NUDT16L1 | nudix (nucleoside diphosphate linked moiety X)-type motif 16-like 1 | 0.184 | 0.043 |
| 40 | OLFML2B | olfactomedin-like 2B | 0.437 | 0.037 |
| 41 | PIN1 | peptidylprolyl cis/trans isomerase, NIMA-interacting 1 | 0.467 | 0.030 |
| 42 | RAB34 | RAB34, member RAS oncogene family | 0.206 | 0.027 |
| 43 | RAB5C | RAB5C, member RAS oncogene family | 0.814 | 0.047 |
| 44 | RANBP10 | RAN binding protein 10 | 0.096 | 0.033 |
| 45 | RNASEH2A | ribonuclease H2, subunit A | 0.112 | 0.025 |
| 46 | RPS19BP1 | ribosomal protein S19 binding protein 1 | 0.398 | 0.032 |
| 47 | SERTAD1 | SERTA domain containing 1 | 0.253 | 0.050 |
| 48 | ST3GAL4 | ST3 beta-galactoside alpha-2,3-sialyltransferase 4 | 0.546 | 0.033 |
| 49 | SWI5 | SWI5 recombination repair homolog (yeast) | 0.187 | 0.039 |
| 50 | TBCC | tubulin folding cofactor C | 0.12 | 0.011 |
| 51 | TCEB2 | transcription elongation factor B (SIII), polypeptide 2 (18kDa, elongin B) | 0.084 | 0.049 |
| 52 | THAP3 | THAP domain containing, apoptosis associated protein 3 | 0.093 | 0.041 |
| 53 | THAP7 | THAP domain containing 7 | 0.149 | 0.022 |
| 54 | TMEM160 | transmembrane protein 160 | 0.259 | 0.013 |
| 55 | TNFRSF12A | tumor necrosis factor receptor superfamily, member 12A | 1.431 | 0.039 |
| 56 | TPM4 | tropomyosin 4 | 3.132 | 0.034 |
| 57 | TSPAN4 | tetraspanin 4 | 0.626 | 0.045 |
| 58 | UCK1 | uridine-cytidine kinase 1 | 0.099 | 0.043 |
| 59 | VCAN | versican | 0.47 | 0.017 |
| 60 | YIPF2 | Yip1 domain family, member 2 | 0.298 | 0.037 |
| 61 | ZNF593 | zinc finger protein 593 | 0.162 | 0.039 |
|  |  |  |  |  |
|  |  |  |  |  |
|  |  | **Genes responsive to Fat Mass and Weight Gain** |  |  |
|  | **Symbol** | **Entrez Gene Name** | **Log Ratio** | **p-value** |
|  |  |  |  |  |
| 1 | ADO | 2-aminoethanethiol (cysteamine) dioxygenase | 0.077 | 0.030 |
| 2 | AMPD2 | adenosine monophosphate deaminase 2 | 0.094 | 0.034 |
| 3 | APBA3 | amyloid beta (A4) precursor protein-binding, family A, member 3 | 0.128 | 0.010 |
| 4 | APRT | adenine phosphoribosyltransferase | 0.281 | 0.037 |
| 5 | ARFGAP1 | ADP-ribosylation factor GTPase activating protein 1 | 0.171 | 0.012 |
| 6 | ARFIP2 | ADP-ribosylation factor interacting protein 2 | 0.15 | 0.037 |
| 7 | B4GALT2 | UDP-Gal:betaGlcNAc beta 1,4- galactosyltransferase, polypeptide 2 | 0.159 | 0.037 |
| 8 | BCL2L12 | BCL2-like 12 (proline rich) | 0.162 | 0.023 |
| 9 | BCL7C | B-cell CLL/lymphoma 7C | 0.536 | 0.014 |
| 10 | C11orf68 | chromosome 11 open reading frame 68 | 0.24 | 0.024 |
| 11 | C16orf58 | chromosome 16 open reading frame 58 | 0.216 | 0.026 |
| 12 | C1orf198 | chromosome 1 open reading frame 198 | 0.225 | 0.015 |
| 13 | C6orf57 | chromosome 6 open reading frame 57 | -0.122 | 0.024 |
| 14 | CADM4 | cell adhesion molecule 4 | 0.625 | 0.014 |
| 15 | CCBL2 | cysteine conjugate-beta lyase 2 | -0.147 | 0.013 |
| 16 | CCDC90B | coiled-coil domain containing 90B | -0.139 | 0.016 |
| 17 | CD14 | CD14 molecule | 0.729 | 0.034 |
| 18 | CD151 | CD151 molecule (Raph blood group) | 0.816 | 0.018 |
| 19 | CDC20 | cell division cycle 20 | 0.143 | 0.031 |
| 20 | CDK2AP2 | cyclin-dependent kinase 2 associated protein 2 | 0.644 | 0.021 |
| 21 | CDKN2C | cyclin-dependent kinase inhibitor 2C (p18, inhibits CDK4) | 0.209 | 0.008 |
| 22 | CERCAM | cerebral endothelial cell adhesion molecule | 0.215 | 0.024 |
| 23 | CGREF1 | cell growth regulator with EF-hand domain 1 | 0.105 | 0.032 |
| 24 | CGRRF1 | cell growth regulator with ring finger domain 1 | -0.132 | 0.043 |
| 25 | CLEC11A | C-type lectin domain family 11, member A | 0.35 | 0.007 |
| 26 | CLNS1A | chloride channel, nucleotide-sensitive, 1A | -0.155 | 0.049 |
| 27 | CMSS1 | cms1 ribosomal small subunit homolog (yeast) | -0.043 | 0.039 |
| 28 | COLEC11 | collectin sub-family member 11 | 0.694 | 0.006 |
| 29 | COTL1 | coactosin-like F-actin binding protein 1 | 0.482 | 0.027 |
| 30 | CSRNP2 | cysteine-serine-rich nuclear protein 2 | 0.096 | 0.033 |
| 31 | DDX56 | DEAD (Asp-Glu-Ala-Asp) box helicase 56 | 0.178 | 0.026 |
| 32 | DKK3 | dickkopf WNT signaling pathway inhibitor 3 | 1.086 | 0.010 |
| 33 | DYNC2LI1 | dynein, cytoplasmic 2, light intermediate chain 1 | -0.15 | 0.031 |
| 34 | EBAG9 | estrogen receptor binding site associated, antigen, 9 | -0.108 | 0.034 |
| 35 | EFEMP1 | EGF containing fibulin-like extracellular matrix protein 1 | 1.716 | 0.006 |
| 36 | EIF2B3 | eukaryotic translation initiation factor 2B, subunit 3 gamma, 58kDa | -0.182 | 0.025 |
| 37 | ELK1 | ELK1, member of ETS oncogene family | 0.329 | 0.020 |
| 38 | ETV6 | ets variant 6 | 0.079 | 0.036 |
| 39 | EXOSC4 | exosome component 4 | 0.246 | 0.010 |
| 40 | F2R | coagulation factor II (thrombin) receptor | 0.593 | 0.019 |
| 41 | F2RL2 | coagulation factor II (thrombin) receptor-like 2 | 0.469 | 0.009 |
| 42 | FAM160A2 | family with sequence similarity 160, member A2 | 0.056 | 0.048 |
| 43 | FLAD1 | flavin adenine dinucleotide synthetase 1 | 0.049 | 0.012 |
| 44 | FOLR2 | folate receptor 2 (fetal) | 0.186 | 0.032 |
| 45 | FRMD8 | FERM domain containing 8 | 0.093 | 0.016 |
| 46 | GORASP1 | golgi reassembly stacking protein 1, 65kDa | 0.133 | 0.033 |
| 47 | GPANK1 | G patch domain and ankyrin repeats 1 | 0.188 | 0.023 |
| 48 | GPKOW | G patch domain and KOW motifs | 0.135 | 0.001 |
| 49 | HDHD2 | haloacid dehalogenase-like hydrolase domain containing 2 | -0.106 | 0.026 |
| 50 | HMG20B | high mobility group 20B | 0.428 | 0.013 |
| 51 | HNRNPA3 | heterogeneous nuclear ribonucleoprotein A3 | 0.059 | 0.035 |
| 52 | HOMER3 | homer homolog 3 (Drosophila) | 0.333 | 0.004 |
| 53 | IER5 | immediate early response 5 | 0.094 | 0.004 |
| 54 | IGFBP1 | insulin-like growth factor binding protein 1 | -3.578 | 0.013 |
| 55 | IGFBP2 | insulin-like growth factor binding protein 2, 36kDa | 4.811 | 0.025 |
| 56 | ITM2C | integral membrane protein 2C | 0.952 | 0.012 |
| 57 | ITPKC | inositol-trisphosphate 3-kinase C | 0.07 | 0.008 |
| 58 | LASP1 | LIM and SH3 protein 1 | 0.524 | 0.047 |
| 59 | LOXL2 | lysyl oxidase-like 2 | 0.544 | 0.032 |
| 60 | LYSMD1 | LysM, putative peptidoglycan-binding, domain containing 1 | 0.092 | 0.014 |
| 61 | MARCKS | myristoylated alanine-rich protein kinase C substrate | 0.242 | 0.017 |
| 62 | MAZ | MYC-associated zinc finger protein (purine-binding transcription factor) | 0.296 | 0.047 |
| 63 | MBD3 | methyl-CpG binding domain protein 3 | 0.255 | 0.027 |
| 64 | METAP1D | methionyl aminopeptidase type 1D (mitochondrial) | -0.109 | 0.002 |
| 65 | MEX3D | mex-3 RNA binding family member D | 0.094 | 0.029 |
| 66 | MFSD11 | major facilitator superfamily domain containing 11 | -0.112 | 0.044 |
| 67 | MICAL2 | microtubule associated monooxygenase, calponin and LIM domain containing 2 | 0.183 | 0.027 |
| 68 | MPRIP | myosin phosphatase Rho interacting protein | 0.524 | 0.044 |
| 69 | MRRF | mitochondrial ribosome recycling factor | -0.138 | 0.043 |
| 70 | MTERFD1 | MTERF domain containing 1 | -0.115 | 0.040 |
| 71 | MTX1 | metaxin 1 | 0.071 | 0.044 |
| 72 | MZT2B | mitotic spindle organizing protein 2B | 1.476 | 0.006 |
| 73 | NAPA | N-ethylmaleimide-sensitive factor attachment protein, alpha | 0.487 | 0.014 |
| 74 | NARF | nuclear prelamin A recognition factor | 0.053 | 0.030 |
| 75 | NARFL | nuclear prelamin A recognition factor-like | 0.071 | 0.025 |
| 76 | NASP | nuclear autoantigenic sperm protein (histone-binding) | 0.07 | 0.042 |
| 77 | NT5DC2 | 5'-nucleotidase domain containing 2 | 0.383 | 0.010 |
| 78 | NTN4 | netrin 4 | 0.108 | 0.047 |
| 79 | NUDT1 | nudix (nucleoside diphosphate linked moiety X)-type motif 1 | 0.223 | 0.010 |
| 80 | PAGR1 | PAXIP1 associated glutamate-rich protein 1 | 0.148 | 0.016 |
| 81 | PAWR | PRKC, apoptosis, WT1, regulator | 0.077 | 0.048 |
| 82 | PKDCC | protein kinase domain containing, cytoplasmic | 0.277 | 0.048 |
| 83 | PMEPA1 | prostate transmembrane protein, androgen induced 1 | 0.166 | 0.049 |
| 84 | PPP1R16A | protein phosphatase 1, regulatory subunit 16A | 0.073 | 0.027 |
| 85 | PPT1 | palmitoyl-protein thioesterase 1 | -0.413 | 0.031 |
| 86 | PQBP1 | polyglutamine binding protein 1 | 0.227 | 0.043 |
| 87 | PRKX | protein kinase, X-linked | 0.258 | 0.040 |
| 88 | PRMT5 | protein arginine methyltransferase 5 | 0.166 | 0.002 |
| 89 | RAB33B | RAB33B, member RAS oncogene family | 0.096 | 0.004 |
| 90 | RCN3 | reticulocalbin 3, EF-hand calcium binding domain | 0.399 | 0.003 |
| 91 | RHOBTB1 | Rho-related BTB domain containing 1 | 0.239 | 0.015 |
| 92 | RNF121 | ring finger protein 121 | 0.102 | 0.006 |
| 93 | RRP9 | ribosomal RNA processing 9, small subunit (SSU) processome component, homolog (yeast) | 0.067 | 0.020 |
| 94 | SCNM1 | sodium channel modifier 1 | 0.133 | 0.016 |
| 95 | SCOC | short coiled-coil protein | -0.17 | 0.024 |
| 96 | SDC2 | syndecan 2 | 2.103 | 0.009 |
| 97 | SDF2L1 | stromal cell-derived factor 2-like 1 | 0.335 | 0.010 |
| 98 | SELENBP1 | selenium binding protein 1 | 0.336 | 0.024 |
| 99 | SERPINH1 | serpin peptidase inhibitor, clade H (heat shock protein 47), member 1, (collagen binding protein 1) | 4.218 | 0.010 |
| 100 | SFRP4 | secreted frizzled-related protein 4 | 1.647 | 0.004 |
| 101 | SH3BP5L | SH3-binding domain protein 5-like | 0.12 | 0.033 |
| 102 | SLC25A29 | solute carrier family 25 (mitochondrial carnitine/acylcarnitine carrier), member 29 | 0.165 | 0.045 |
| 103 | SLC39A13 | solute carrier family 39 (zinc transporter), member 13 | 0.32 | 0.048 |
| 104 | SMIM14 | small integral membrane protein 14 | -0.27 | 0.005 |
| 105 | SMUG1 | single-strand-selective monofunctional uracil-DNA glycosylase 1 | 0.11 | 0.035 |
| 106 | SNX8 | sorting nexin 8 | 0.08 | 0.020 |
| 107 | SPESP1 | sperm equatorial segment protein 1 | -0.099 | 0.010 |
| 108 | STX5 | syntaxin 5 | 0.165 | 0.018 |
| 109 | TANC1 | tetratricopeptide repeat, ankyrin repeat and coiled-coil containing 1 | 0.084 | 0.029 |
| 110 | TDRD7 | tudor domain containing 7 | -0.053 | 0.004 |
| 111 | TEN1 | TEN1 CST complex subunit | 0.206 | 0.003 |
| 112 | TGFBI | transforming growth factor, beta-induced, 68kDa | 0.23 | 0.048 |
| 113 | TMCO6 | transmembrane and coiled-coil domains 6 | 0.05 | 0.001 |
| 114 | TMEM219 | transmembrane protein 219 | 0.291 | 0.015 |
| 115 | TMEM222 | transmembrane protein 222 | 0.126 | 0.035 |
| 116 | TMEM60 | transmembrane protein 60 | -0.204 | 0.040 |
| 117 | TSEN54 | TSEN54 tRNA splicing endonuclease subunit | 0.085 | 0.045 |
| 118 | TYW3 | tRNA-yW synthesizing protein 3 homolog (S. cerevisiae) | -0.056 | 0.016 |
| 119 | U2AF2 | U2 small nuclear RNA auxiliary factor 2 | 0.554 | 0.049 |
| 120 | ULBP3 | UL16 binding protein 3 | 0.163 | 0.045 |
| 121 | URI1 | URI1, prefoldin-like chaperone | -0.097 | 0.004 |
| 122 | USP25 | ubiquitin specific peptidase 25 | -0.034 | 0.017 |
| 123 | UXT | ubiquitously-expressed, prefoldin-like chaperone | 0.534 | 0.017 |
| 124 | WDR18 | WD repeat domain 18 | 0.124 | 0.040 |
| 125 | WDR34 | WD repeat domain 34 | 0.131 | 0.033 |
| 126 | WRAP53 | WD repeat containing, antisense to TP53 | 0.072 | 0.028 |
| 127 | WRNIP1 | Werner helicase interacting protein 1 | 0.122 | 0.032 |
| 128 | YY1 | YY1 transcription factor | 0.194 | 0.008 |
| 129 | ZNF511 | zinc finger protein 511 | 0.224 | 0.003 |
|  |  |  |  |  |
|  |  | **Genes responsive exclusively to High Fat High Fructose Diet and Fat Mass** |  |  |
|  | **Symbol** | **Entrez Gene Name** | **Fold Change** | **p-value** |
|  |  |  |  |  |
| 1 | ACTR10 | actin-related protein 10 homolog (S. cerevisiae) | -2.83 | 0.029 |
| 2 | HYAL1 | hyaluronoglucosaminidase 1 | 3 | 0.038 |
| 3 | IKZF5 | IKAROS family zinc finger 5 (Pegasus) | 2 | 0.011 |
| 4 | MRPS9 | mitochondrial ribosomal protein S9 | -8.04 | 0.048 |
| 5 | PSMD7 | proteasome (prosome, macropain) 26S subunit, non-ATPase, 7 | -2.87 | 0.044 |
| 6 | RAI14 | retinoic acid induced 14 | 8 | 0.041 |
| 7 | TACC1 | transforming, acidic coiled-coil containing protein 1 | 3 | 0.022 |
| 8 | TOP3B | topoisomerase (DNA) III beta | 2 | 0.038 |
| 9 | TSN | translin | -4 | 0.043 |
| 10 | VAMP4 | vesicle-associated membrane protein 4 | -3.54 | 0.028 |
|  |  |  |  |  |
|  |  |  |  |  |
|  |  | **Gene responsive exclusively to High Fat High Fructose Diet and Weight Gain** |  |  |
|  | **Symbol** | **Entrez Gene Name** | **Fold Change** | **p-value** |
|  |  |  |  |  |
| 1 | C8orf82 | chromosome 8 open reading frame 82 | 4 | 4.98E-02 |
|  |  |  |  |  |
|  |  |  |  |  |
|  |  | **Genes responsive to High Fat High Fructose Diet, Fat Mass and Weight Gain** |  |  |
|  | **Symbol** | **Entrez Gene Name** | **Fold Change** | **p-value** |
|  |  |  |  |  |
| 1 | BBS5 | Bardet-Biedl syndrome 5 | -27.36 | 0.011 |
| 2 | BIN3 | bridging integrator 3 | 3 | 0.014 |
| 3 | LACTB2 | lactamase, beta 2 | -53.6 | 0.034 |
| 4 | PRR24 | proline rich 24 | 3 | 0.032 |
| 5 | PYCR1 | pyrroline-5-carboxylate reductase 1 | 4 | 0.043 |
| 6 | RAP1B | RAP1B, member of RAS oncogene family | -8.6 | 0.041 |
| 7 | RRP7A | ribosomal RNA processing 7 homolog A (S. cerevisiae) | 7 | 0.044 |
| 8 | SGSM3 | small G protein signaling modulator 3 | 4 | 0.032 |
