# Supplementary material for: Joint MiRNA/mRNA Expression Profiling Reveals Changes Consistent with Development of Dysfunctional Corpus Luteum after Weight Gain
Source: PLoS One. 2015 Aug 10;10(8):e0135163. doi: 10.1371/journal.pone.0135163 (PMC4530955; doi:10.1371/journal.pone.0135163)
Supplement: S4 Table — (DOCX) [file pone.0135163.s007.docx]

| **S4 Table. MRNA Quality Control Report** | | | | | |
| --- | --- | --- | --- | --- | --- |
| Sample | Raw sequences reads | Reads after QC (%) | % | Mapped reads (%) | % |
| 1150_10_9 | 68,671,250 | 58,851,394 | 85.7 | 55,026,808 | 93.5 |
| 1204_6_28 | 63,296,440 | 52,599,340 | 83.1 | 48,587,301 | 92.4 |
| 1172_8_31 | 61,808,258 | 54,015,752 | 87.4 | 50,579,218 | 93.6 |
| 1172_8_3 | 63,569,546 | 59,167,174 | 93.1 | 55,632,460 | 94 |
| 1150_11_14 | 88,238,462 | 69,151,782 | 78.4 | 64,797,019 | 93.7 |
| 1204_7_26 | 88,013,078 | 81,706,518 | 92.8 | 77,224,281 | 94.5 |
| 1207_5_21 | 77,338,634 | 69,798,736 | 90.3 | 64,671,172 | 92.7 |
| 1207_6_6 | 91,509,916 | 84,537,992 | 92.4 | 79,632,903 | 94.2 |
| 1227_8_21 | 74,126,870 | 60,123,526 | 81.1 | 56,559,983 | 94.1 |
| 1208_10_19 | 101,609,422 | 93,142,872 | 91.7 | 88,055,381 | 94.5 |
| 1208_9_26 | 94,345,914 | 87,560,200 | 92.8 | 82,783,189 | 94.5 |
| 1227_9_6 | 95,599,226 | 85,573,620 | 89.5 | 80,914,552 | 94.6 |
| 1266_9_26 | 84,839,590 | 76,014,372 | 89.6 | 71,619,669 | 94.2 |
| 1286_6_18 | 77,057,112 | 70,627,816 | 91.7 | 66,601,001 | 94.3 |
| 1266_10_27 | 97,186,632 | 82,066,370 | 84.4 | 76,869,187 | 93.7 |
| 1286_7_25 | 79,182,022 | 74,139,944 | 93.6 | 70,082,641 | 94.5 |
| 1302_6_15 | 76,997,576 | 67,019,852 | 87 | 62,806,816 | 93.7 |
| 1377_10_5 | 74,644,526 | 68,748,522 | 92.1 | 65,010,749 | 94.6 |
| 1302_7_23 | 81,658,658 | 71,989,026 | 88.2 | 67,920,319 | 94.3 |
| 1377_10_3 | 83,486,818 | 78,562,198 | 94.1 | 74,153,418 | 94.4 |
