# Supplementary material for: Joint MiRNA/mRNA Expression Profiling Reveals Changes Consistent with Development of Dysfunctional Corpus Luteum after Weight Gain
Source: PLoS One. 2015 Aug 10;10(8):e0135163. doi: 10.1371/journal.pone.0135163 (PMC4530955; doi:10.1371/journal.pone.0135163)
Supplement: S5 Table — (DOCX) [file pone.0135163.s008.docx]

| **S5 Table. MiRNA Quality Control Report** | | | |
| --- | --- | --- | --- |
| Sample | Raw sequences reads | Reads after QC | % |
| 1227_9-6 | 20,589,839 | 3,808,608 | 18.5 |
| 1150_10-9 | 21,917,654 | 4,240,663 | 19.3 |
| 1302_6-15 | 28,623,317 | 4,473,488 | 15.6 |
| 1150_11-14 | 21,046,189 | 3,818,310 | 18.1 |
| 1302_7-23 | 25,690,380 | 4,315,700 | 16.8 |
| 1172_8-31 | 26,684,089 | 7,558,302 | 28.3 |
| 1172_8-3 | 20,472,846 | 3,292,885 | 16.1 |
| 1377_10-3 | 32,700,657 | 4,854,037 | 14.8 |
| 1266_9-26 | 27,629,490 | 4,631,331 | 16.8 |
| 1204_6-28 | 20,084,317 | 3,010,487 | 15 |
| 1377_10-5 | 19,816,775 | 3,582,471 | 18.1 |
| 1204_7-26 | 20,989,381 | 3,446,069 | 16.4 |
| 1207_5-21 | 34,634,025 | 5,404,645 | 15.6 |
| 1286_6-18 | 16,132,447 | 3,337,452 | 20.7 |
| 1207_6-6 | 23,893,973 | 5,270,562 | 22.1 |
| 1208_10-19 | 10,922,016 | 2,476,890 | 22.7 |
| 1208_9-26 | 22,624,004 | 4,221,108 | 18.7 |
| 1227_8-21 | 30,593,669 | 5,350,958 | 17.5 |
| 1266_10-27 | 24,766,915 | 4,458,776 | 18 |
| 1286_7-25 | 2,951,200 | 230,036 | 7.8 |
